# Batoid Abundances, Spatial Distribution, and Life History Traits in the Strait of Sicily (Central Mediterranean Sea): Bridging a Knowledge Gap through Three Decades of Survey

**DOI:** 10.3390/ani11082189

**Published:** 2021-07-23

**Authors:** Michele Luca Geraci, Sergio Ragonese, Danilo Scannella, Fabio Falsone, Vita Gancitano, Jurgen Mifsud, Miriam Gambin, Alicia Said, Sergio Vitale

**Affiliations:** 1 Geological and Environmental Sciences (BiGeA)–Marine Biology and Fisheries Laboratory, Department of Biological, University of Bologna, Viale Adriatico 1/n, 61032 Fano, PU, Italy; micheleluca.geraci2@unibo.it; 2 Institute for Marine Biological Resources and Biotechnology (IRBIM), National Research Council–CNR, Via Luigi Vaccara, 61, 91026 Mazara del Vallo, TP, Italy; danilo.scannella@irbim.cnr.it (D.S.); fabio.falsone@irbim.cnr.it (F.F.); vita.gancitano@cnr.it (V.G.); sergio.vitale@cnr.it (S.V.); 3 Department of Fisheries and Aquaculture, Ministry for Agriculture, Fisheries and Animal Rights (MAFA), Ghammieri Government Farm, Triq l-Ingiered, Malta; jurgen.a.mifsud@gov.mt (J.M.); miriam.gambin@gov.mt (M.G.); alicia.bugeja-said@gov.mt (A.S.)

**Keywords:** Mediterranean Sea, bottom trawl survey, spatial distribution, length at first maturity, Linfinity, sex ratio, length–weight relationship

## Abstract

**Simple Summary:**

Batoid species are cartilaginous fish commonly known as rays, but they also include stingrays, electric rays, guitarfish, skates, and sawfish. These species are very sensitive to fishing, mainly because of their slow growth rate and late maturity; therefore, they need to be adequately managed. Regrettably, information on life history traits (e.g., length at first maturity, sex ratio, and growth) and abundance are still scarce, particularly in the Mediterranean Sea. In this regard, the present study focuses on the Strait of Sicily (Central Mediterranean) and aims to improve knowledge gained through scientific survey data. In particular, abundance data, spatial distribution, and some life history traits are herein presented. In the investigated area, the biomass trends of the batoids indicated a slight recovery even if few species showed a depletion. Considering the importance of this taxon for maintaining the marine ecosystem equilibrium, management measures are desirable.

**Abstract:**

Batoid species play a key role in marine ecosystems but unfortunately they have globally declined over the last decades. Given the paucity of information, abundance data and the main life history traits for batoids, obtained through about three decades of bottom trawl surveys, are presented and discussed. The surveys were carried out in two areas of the Central Mediterranean (South of Sicily and Malta Island), in a timeframe ranging from 1990 to 2018. Excluding some batoids, the abundance trends were stable or increasing. Only *R. clavata*, *R. miraletus*, and *D. oxyrinchus* showed occurrence and abundance indexes notable enough to carry out more detailed analysis. In particular, spatial distribution analysis of these species highlighted the presence of two main hotspots in Sicilian waters whereas they seem more widespread in Malta. The lengths at first maturity (L_50_) were 695 and 860, 635 and 574, and 364 and 349 mm total length (TL), respectively, for females and males of *D. oxyrinchus*, *R. clavata*, and *R. miraletus*. The asymptotic lengths (L∞) and the curvature coefficients (K) were 1365 and 1240 (K = 0.11 and 0.26), 1260 and 1100 (K = 0.16 and 0.26), and 840 and 800 mm TL (K = 0.36 and 0.41), respectively, for females and males of *D. oxyrinchus*, *R. clavata*, and *R. miraletus*. The lack of detailed quantitative historical information on batoids of Sicily and Malta does not allow to analytically judge the current status of the stocks, although the higher abundance of some species within Malta raises some concern for the Sicilian counterpart. In conclusion, suitable actions to protect batoids in the investigated area are recommended.

## 1. Introduction

Batoidea is an infraclass of cartilaginous fish commonly known as batoids or rays, but it also includes stingrays, electric rays, guitarfish, skates, and sawfish. Batoid fishes are moderately to greatly flattened and are distinguished from other elasmobranchs (non-batoid sharks) by their ventral gill slits, their lack of an anal fin, and having the pectoral fins connected to the sides of the head and trunk to form a disk [1,2]. In the Mediterranean Sea (herein Mediterranean), at least 38 species of batoid fishes have been reported [3]. However, long-term sources of information to assess the batoids’ exploitation status are very rare in this region [4,5]. Up until the 1980s, these animals were considered as nuisance species having low economic value to Mediterranean fishers, and consequently most of them were discarded. Therefore, these species were neither recorded in the official landing statistics nor in the first experimental surveys (see, in [5,6]). Thereafter, facing the decline in the more productive target bony and shellfish species and the increasing demand of markets, fishers began to retain and land large batoids, returning to the sea only the small or damaged specimens [7].

The lack of significant and representative historical data (reflecting also the misclassification of these species) makes it almost impossible to gauge the current standing stock condition and to detect depletion (if any) [5,6,8]. Moreover, there is evidence that Mediterranean batoid stocks (as well as their oceanic counterparts) are extremely vulnerable to fishing intensity, even when the resulting fishing mortality remains at a low level and they are exposed to relatively short periods of exploitation [9,10]. As a result, nowadays there is general agreement on the generalized rarefaction and bad exploitation status of almost all Mediterranean batoid stocks (see, in [11,12]), even though few quantitative assessments have been conducted. Furthermore, considering that they play an important ecological role as they are meso-predator [13], and, being very sensitive to any change in the ecosystem, they are often used as biological indicators [14,15,16]. In the last decades, the IUCN (International Union for Conservation of Nature) has focused on these vulnerable taxa and provided a relative estimation of the likelihood of extinction, which is summarized in the “European Red list of Fishes” (see, in [11,17]). Regarding the GFCM (General Fisheries Commission for the Mediterranean) geographical sub-areas (GSA16 and GSA15), named South of Sicily and Malta Island, information has been made available through a standardized scientific international program launched in 1994, the MEDITS (International bottom trawl survey in the Mediterranean [18,19]). In addition, the MEDITS surveys were integrated between 1990 and 2006 by the homologous Italian surveys program called GRUND (GRUppo Nazionale Demersali).

Only scattered data on distribution and abundance, and limited information on growth and unit stock identification, have been published for the Strait of Sicily sensu Jereb et [20]. In particular, contributions on batoids can be found for the Sicilian waters [21,22,23,24,25,26,27], the Malta Island [28], and for Tunisian waters [29,30,31,32,33,34,35,36].

In this context, the aim of this paper is to provide a summary of the current knowledge on batoid fishes occurring in GSA16 and GSA15. This will be done through the use of the frequency of occurrences, abundance indices, and some relevant biological information provided through the MEDITS and GRUND surveys, performed in a wide time interval following, as closely as possible, the same methodologies. The present work provides a basic tool for implementing a specific management plan for these endangered species.

## 2. Materials and Methods

### 2.1. Study Area and Sampling Methodology

**Figure 1:**
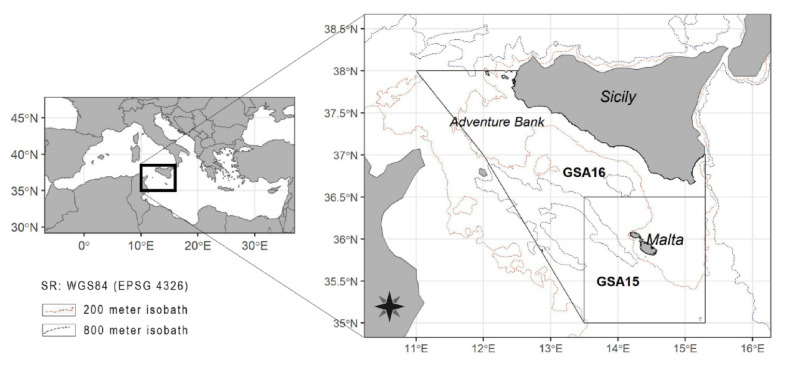
The study area with the two considered Geographical Sub-Areas: South of Sicily (GSA16) and Malta Island (GSA15). Red and blue dashed lines denote the 200 and 800 m isobaths, respectively.

The analyzed data refer to a wide area located between the Southern coasts of Sicily and the Northern coasts of Africa. According to the GFCM classification [37], the study areas are (i) South of Sicily, GSA16, and (ii) Malta Island, GSA15 (Figure 1).

A database collecting about three decades of experimental (scientific) bottom trawl surveys in GSA16 and GSA15 was used. For the surveys, the analyzed data refer to the batoids directly sampled mainly in spring and summer during the MEDITS survey from 1994 to 2018 in GSA16 (herein MEDITS16) and from 2005 to 2018 in GSA15 (herein MEDITS15), and in autumn during the GRUND survey from 1990 to 2006 in both GSAs (herein GRUND15 and GRUND16, survey carried out respectively in the GSA15 and GSA16). The changes to survey protocols/specifications over the years are documented in the manual [18] and descriptive papers [19,38,39,40]. In particular, the foreseen average sampling rate was one station per 60 square nautical miles in both areas. As closely as possible, the same stations were visited each year. Within MEDITS, sampling at sea has always been conducted with the bottom trawl net GOC73 [19,40], whereas in GRUND a typical commercial trawl net, locally called “Mazarese” or “tartana di banco”, was used [38,39]. Specifically, the two gears mainly differ in the vertical mouth opening (2.4–2.9 in MEDITS vs. 0.6–1.3 m in GRUND), but both gears mount a 20 mm side diamond stretched mesh in the cod end. In both surveys, the haul durations were 30′ and 60′ at stations between 10 and 200 m (shelf) and 201–800 m (slope), respectively. MEDITS16 and MEDITS15 have been carried out consistently, with MEDITS15 starting in 2005, whereas GRUND was not carried out in 1993 and 1999 because of administrative constraints (Tables S1 and S2). The stations were distributed through a stratified sampling scheme with random drawings inside each stratum. The stratification criterion adopted was the depth, with the following bathymetric limits being 10 to 50 m (a stratum), 51 to 100 m (b stratum), 101 to 200 m (c stratum), 201 to 500 m (d stratum), and 501 to 800 m (e stratum). However, in order to simplify the presentation of abundance indices, the previous microstrata were also pooled into two macrostrata: shelf (10–200 m) and slope (201–800 m).

### 2.2. Abundance Data and Biological Parameter Analyses

The biological samples, from sampling at the sea, were identified, frozen, and brought to the laboratories for successive biometric (Total Length, TL, in mm), gravimetric (Total body weight, W, in g), and, after dissection, reproductive analyses by distinguishing macroscopically the sex (F, females; M, males) and maturity stages. The data collected and elaborated through the surveys were presented as follows (the symbols refer to the work in [41], if not otherwise specified):

(a)  frequency of occurrence as percentage of positive hauls (f%) and survey (s%);
(b)  two abundance indices: in weight, Biomass Index (BI; kg/km^2^), and number, Density Index (DI; N/km^2^), expressed as grand mean (the mean of the mean) with their relative standard error; f% and both abundance indices were estimated for the continental shelf (10–200 m), slope (200–800 m), and overall (10–800 m) depth stratum;
(c)  depth presence among the identified five microstrata;
(d)  correlation among survey abundance indexes and years were assessed by GSA, species, and macrostratum, by computing the nonparametric Spearman linear rank coefficient;
(e)  overall standing stock expressed in weight (tons) and number (thousand);
(f)  biological information in terms of overall sex ratio (sex ratio = F/M), median length, and length–weight relationship (LWR, power function; *k* will indicate the steepness; *b* will indicate the positive (*b* > 3) or negative (*b* < 3) allometric coefficient; isometric when *b* = 3) only from MEDITS16 because of the longer time series.

Sex ratio deviations from the expected 1:1 were checked by applying the nonparametric Chi-square test [42]; *p* < 0.05 was considered statistically significant.

In addition, for the most representative and abundant species, identified according to the few, although arbitrary, thresholds (f% ≥ 10 and 50 tons in standing stock at least in two surveys), the following biological parameters were also estimated for females and males from MEDITS16:

(i)  length at first sexual maturity (L_50_, also known as at onset of sexual maturity), i.e., the length at which 50% of specimens resulted mature (stage 3a onward according to MEDITS gonadic scale [18]), or in case of lack of fit of the logistic using the median length of the 3a stage as a proxy of L_50_ [43];
(ii)  Length–Frequency Distribution (LFD) “stability” in the different years (performed via the Kolmogorov–Smirnov test);
(iii)  L∞ (the infinite/asymptotic length at which the average growth rate of the oldest cohort becomes close to zero) and K (Brody or curvature coefficient), both related to the von Bertalanffy growth function or;
(iv)  Z/K ratio by sex [44] at 95% confidence interval, where Z denotes the instantaneous rate of total mortality (as fishing, F, plus natural mortalities, M). The previous parameters were estimated using the ELEFAN (Electronic Length Frequencies Analysis) and Powell–Wetherall procedures in the TropFishR package [45]. All the size measurements were expressed as the Total Length (TL) in mm.

Furthermore, the life history traits estimated in the present study were compared to those of the other Mediterranean studies (Table S3).

Finally, yearly DI plots of the surveys were represented. Differences between the means of the DI, BI, and frequency of occurrence (%) in the two considered GSA for these species were tested using the nonparametric Wilcoxon’s signed rank test (or Mann–Whitney *U* test) for surveys datasets; *p* < 0.05 was considered statistically significant. All the data were checked for normality using the empirical distribution of the data (the histogram), Shapiro–Wilk test, and qqplot (quantile-quantile plot).

### 2.3. Spatial Analysis

Geo-statistical analysis [46,47] was applied to the DI in order to obtain the horizontal distribution map of the three species identified from the above prefixed thresholds, i.e., *D. oxyrinchus*, *R. clavata*, and *R miraletus*. Overall, the datasets used consisted of 2750 and 1737 hauls from MEDITS and GRUND, respectively. Considering that geo-statistic techniques use spatial interpolation procedures to obtain spatially continuous variables from isolated station measurements [48,49], to avoid any sampling area “weighing” the estimates more than another location with few hauls, a data manipulation series was carried out. First, a 4 × 4 nautical mile grid covering the whole study area (GSA15 and 16) was constructed (1689 cells), and then a WGS84 UTM 33N (World Geographic System 1984, Universal Transverse Mercator 33 Nord) projected coordinate system was set. Second, for each survey, sampled stations within each cell were managed in order to determine a new point with geographical coordinates that minimized the Euclidean distance of all sampled stations within the same cell, where the median DI value was used. At the end of this step, the new points were 421 and 435 from MEDITS and GRUND, respectively. However, the reduction in the number of stations requires a minimum of 30–50 pairs of points to ensure statistical consistency and representativeness of the sampling space [50]. Therefore, these data were log-transformed (DI + 1) to improve the normality and were used to fit asymptotic models and to compute standard parameters of experimental semivariograms (i.e., range, nugget, and sill). To estimate these parameters, among Gaussian, Exponential, and Spherical models the one reducing the residual sum of squares was selected. Then, following the estimation of variogram parameters, ordinary kriging was applied to map the spatial distribution of the DI for each species and survey using the geoR library [51]. Spatial interpolation was performed using a 0.5 × 0.5 km^2^ grid. In addition, maps of the positive hauls for the three above-mentioned species were provided (Figures S1–S3). All analyses were carried out in R studio, version 3.6.2 [52].

## 3. Results

### 3.1. General

A total of 4575 scientific hauls performed over 25 and 17 years, respectively, for MEDITS and GRUND were analyzed, and the results are summarized in Table 1, Table 2, Table 3 and Table 4. The numbers of batoid species reported during MEDITS were 22 in the South of Sicily (GSA16) and 16 in the Malta Island (GSA15), whereas during GRUND they were 20 (in the South of Sicily (GSA16) and 17 in the Malta Island (GSA15), overall belonging to three orders and four families. From Table 1, Table 2, Table 3 and Table 4, it is possible to notice that only three species, namely, *R. clavata*, *R. miraletus*, and *D. oxyrinchus*, showed values above the prefixed thresholds, (f% > 10; SSw > 50 t at least in two surveys). Excluding these species, in MEDITS16 only *Leucoraja melitensis* (Clark, 1926), *Raja asterias* Delaroche, 1809, *Raja montagui* Fowler, 1910, and *Torpedo marmorata* Risso, 1810 showed overall mean DI higher than 2 N/km^2^ (a very low density), whereas in MEDITS15 *Dasyatis pastinaca* (Linnaeus, 1758), *Myliobatis aquila* (Linnaeus, 1758), *Leucoraja circularis* (Couch, 1838), *L. melitensis*, *R. montagui*, *Raja radula* Delaroche, 1809, and *T. marmorata* showed a higher DI. More worrying, only *L. melitensis*, for GRUND16, and *D. pastinaca* and *L. melitensis*, for GRUND15, exceeded a DI of 2 N/km^2^. It is worth remarking that nine species with a standing stock less than 10 tons might be considered close to local extinction. Synthetic results concerning abundance and life history traits (when available) of the taxa sampled during surveys are herein provided.

#### 3.1.1. Great Torpedo Ray—*Tetronarce nobiliana* (Bonaparte, 1835)

The species is diffused over the investigated area but in scant numbers. Even if it was sampled across all strata, it seemed to inhabit the slope as the preferential macrostratum. In spring–summer, *T. nobiliana* was found with higher frequency of occurrence (2.1%), abundance indices (slope: BI = 0.7 ± 0.4 kg/km^2^, DI = 0.3 ± 0.1 N/km^2^), and standing stock (12.5 ± 6.7 tons, 9 ± 2 thousand) in South of Sicily (Table 1 and Table 2). In fact, in autumn it was sampled more frequently in South of Sicily (1.2%) with the highest values on the slope (Table 3 and Table 4); however, no significant correlations over the years were found (Table 1, Table 2, Table 3 and Table 4). The TL ranged from 115 to 970 mm for females, while for males it ranged from 120 to 610 mm. The LWR parameters were 3.3 × 10*^−^*^5^ (k) and 2.89 (b), thus showing negative allometry, for sex combined. The median length was between 395 and 330 mm (TL) for females and males. The sex ratio was 0.63:1 (X^2^ = 4.84, *p* = 0.02781) (Table S3).

#### 3.1.2. Marbled Electric Ray—*Torpedo marmorata* Risso, 1810

The surveys highlighted its wide spatial, as well as depth, distribution. In fact, this species was recorded in all strata in the spring–summer and autumnal surveys in the South of Sicily, although it seemed to be more abundant on the continental shelf. In spring–summer, the species showed the highest frequency of occurrence (11%) and abundance indices (shelf: BI = 1.8 ± 0.5 kg/km^2^, DI = 6.5 ± 1.3 N/km^2^) in Malta Island, although the standing stock was higher in South of Sicily (20.8 ± 2.5 tons, 69 ± 7 thousand) (Table 1 and Table 2). Similarly, in autumn, it was caught more frequently (10.8%) with slightly higher abundance indices (shelf: BI = 0.8 ± 0.4 kg/km^2^, DI = 1.9 ± 0.6 N/km^2^) in Malta Island, although the standing stock was higher in South of Sicily (9.2 ± 2.5 tons, 29 ± 5 thousand). The abundance indices were positively correlated over the years (on the slope) only in Malta Island (Table 3 and Table 4). The TL ranged from 105 to 495 mm for females and from 100 to 370 mm for males. The median lengths were 430 and 445 mm TL for females and males, respectively. The LWR parameters were 3.3 × 10*^−^*^5^, 1 × 10*^−^*^4^, and 4.5 × 10*^−^*^5^ (k) and 2.96, 2.67, and 2.85 (b), respectively, for females, males, and sex combined. The sex ratio was 0.85:1 (X^2^ = 0.64, *p* = 0.4237) (Table S3).

#### 3.1.3. Common Torpedo—*Torpedo torpedo* (Linnaeus, 1758)

*T. torpedo* has been recorded in a wide bathymetric range from the surface (10 m) up to 800 m. In general, it was more abundant on the slope. In spring-summer, it was caught only in South of Sicily with a frequency of occurrence of 0.8%. Note that, in this area, the highest DI values were observed on the shelf (0.6 ± 0.2 N/km^2^), whilst the highest BI value was recorded on the slope (0.3 ± 0.2 kg/km^2^), suggesting a positive relationship between size/age and depth (i.e., juveniles live in shallower water than adults) (Table 1). In autumn, the species was rarely caught, even though a slightly higher abundance was observed in South of Sicily. No significant correlation between abundance and time was found (Table 3 and Table 4). The TL ranged from 105 to 275 mm for females and from 135 to 355 mm for males. The median lengths were 170 and 190 mm TL for females and males, respectively. The LWR parameters were 8.8 × 10*^−^*^6^ (k) and 3.13 (b) for sex combined. The sex ratio was 1.86:1 (X^2^ = 9, *p* = 0.0027) (Table S3).

#### 3.1.4. Gray Skate—*Dipturus batis* (Linnaeus, 1758)

The surveys indicated few records on the slope, in the e stratum (501–800 m), in Adventure Bank. In spring–summer, it was caught only in GSA16 once in 2011, thus with very low values in terms of frequency of occurrence, BI, and DI (Table 1). In autumn, the highest frequency (0.6%), BI (0.7 ± 0.7 kg/km^2^), and DI (0.2 ± 0.2 N/km^2^) were recorded in Malta Island (Table 3 and Table 4). Very few specimens and biological data were collected. The maximum length was 1260 mm TL (female) (Table S3).

#### 3.1.5. Norwegian Skate—*Dipturus nidarosiensis* (Storm, 1881)

Only a single juvenile male specimen was caught in 2017 in Pantelleria bank during spring–summer survey in South of Sicily (see [53] for details).

#### 3.1.6. Longnosed Skate—*Dipturus oxyrinchus* (Linnaeus, 1758)

**Figure 2:**
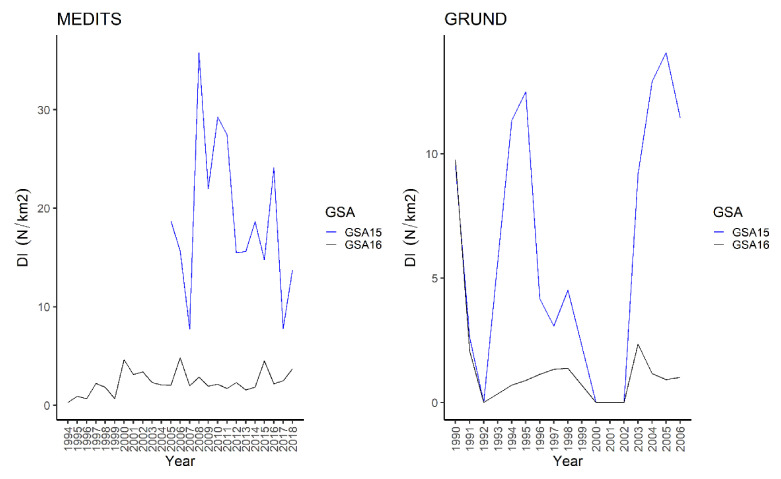
Density Index (DI, N/km^2^) of *Dipturus oxyrinchus* by overall deep interval (10–800 m) in the South of Sicily (GSA16) and Malta Island (GSA15) between 1994 and 2018 during MEDITS–spring summer–(**left**) and between 1990 and 2006 during GRUND–autumn–(**right**).

*D. oxyrinchus* appeared mainly distributed on the slope in surveys. In spring-summer, the highest frequency of occurrence (30%), BI (40.53 ± 5.4 kg/km^2^), and DI (32.3 ± 4.8 N/km^2^) were recorded in Malta Island (Table 1 and Table 2). Similarly, in autumn it was caught more frequently in Malta Island (Table 3 and Table 4). In spring–summer, a slight, although significant, positive correlation was recorded in South of Sicily between the BI over the years on the slope (Table 1). Conversely, a strong, positive correlation was found on the shelf only in autumnal survey in Malta Island (Table 4). As matter of fact, the yearly DI plot showed that a higher index was always recorded from the spring–summer in Malta Island, with a clear peak in 2008. The Mann–Whitney U test showed a significant difference between the DI (W = 196, *p* < 0.001), BI (W = 196, *p* < 0.001), and frequency of occurrence (W = 194, *p* < 0.001) between areas in autumn. Similarly, from the autumnal survey of Malta Island, except for 1990, higher values of DI were recognized with two peaks in 1994 and 2005 (Figure 2).

**Figure 3:**
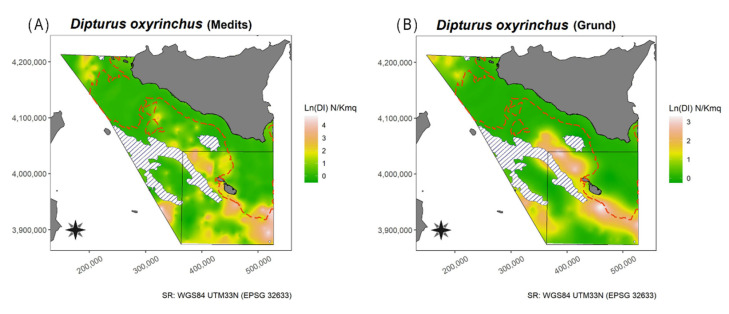
Spatial distribution, in terms of Density Index (DI, N/Km^2^), of *Dipturus oxyrinchus* in the South of Sicily (GSA 16) and Malta island (GSA15) during (**A**) MEDITS–spring summer–and (**B**) GRUND–autumn–surveys. The dashed areas denote depths below 800 m not explored in present surveys.

The Mann–Whitney U test detected a significant difference between the DI (W = 167, *p* = 0.02376), BI (W = 179, *p* = 0.001319), and frequency of occurrence (W = 166, *p* = 0.02644) from the autumnal surveys of both areas. The DI map showed the presence of a little spot located at West of Marettimo Island, and other two bigger spots located in SE Linosa waters and on the NW border of the Malta Island (Figure 3 and Figure S1)with higher occurrences of this species. In these waters the species appeared more widespread, in the NW Malta Island spot and on the slope in the South of Sicily S-SW spots (Figure 3).

The TL ranged from 230 to 1240 mm and 250 to 1100 mm for females and males, respectively. The median length was slightly higher in males (620 mm TL) compared to females (600 mm TL). The LFD showed significant differences between 2010, 2011, and 2014. The LWR parameters were 5.4 × 10*^−^*^7^, 1 × 10*^−^*^6^, and 7.2 × 10*^−^*^7^(k) and 3.29, 3.20, and 3.25 (b), respectively, for females, males, and sex combined. The growth parameters, as estimated through ELEFAN, indicated that females attained a larger L∞ (1365 mm TL) than males (1240 mm TL), while the K results were 0.11 and 0.26 for females and males, respectively. The Z/K values were 1.66 (Confidence Interval: 1.64–1.68) and 1.21 (Confidence Interval: 1.19–1.23) for females and males, respectively. The Z values obtained were 0.18 and 0.31 for females and males, respectively. The estimated L_50_ values (median stage approach) were 695 and 860 mm TL for females and males, respectively. The sex ratio was 1.56:1 (X^2^ = 4.84, *p* = 0.02781) (Table S3).

#### 3.1.7. Sandy Ray—*Leucoraja circularis* (Couch, 1838)

The bathymetric distribution of *L. circularis* was mainly focused on the slope at depths ranging from 100 to 800 m. In spring–summer, the highest values in terms of frequency of occurrence (6%), abundance indices (in slope BI = 41.8 ± 39.8 kg/km^2^, DI = 3.3 ± 1.2 N/km^2^), standing stock (247.5 ± 233.8 tons, 20 ± 7 thousand) were recorded from the Malta Island (Table 1 and Table 2). Similarly, in autumn, the highest values were recorded in Malta Island (Table 3 and Table 4). Very few specimens and biological data were collected. The TL ranged from 310 to 880 mm for females, and from 180 to 750 mm for males (Table S3).

#### 3.1.8. Shagreen Ray—*Leucoraja fullonica* (Linnaeus, 1758)

The present study confirms the rarity of this ray in South of Sicily. In fact, it was never caught in autumn and was very rarely caught in spring-summer. From this study, this species was more frequent (2%) and abundant (standing stock: 2.1 ± 1.5 tons, 3 ± 1 thousand, slope: BI = 0.4 ± 0.2 kg/km^2^, DI = 0.5 ± 0.2 N/km^2^) in Malta Island (Table 1 and Table 2). Very few specimens and no biological data were collected.

#### 3.1.9. Maltese Ray—*Leucoraja melitensis* (Clark, 1926)

The Maltese ray was caught in a wide range of depths, from the surface to 800 m, but it was more common on the slope. During spring–summer, the highest frequency of occurrence (11%) and abundance indices (slope: BI = 3.3 ± 0.6 kg/km^2^, DI = 16.3 ± 2.9 N/km^2^) were observed in Malta Island; however, the highest standing stock values were observed in South of Sicily (26.7 ± 3.0 tons, 132 ± 15 thousand) (Table 1 and Table 2). Conversely, in autumn, the species was more frequent (4.9%) and abundant in the South of Sicily (slope: BI = 0.7 ± 0.2 kg/km^2^; DI = 3.9 ± 1.4 N/km^2^) (Table 3 and Table 4). A slight, positive correlation was observed from the spring-summer survey in South of Sicily over the years in the shelf (Table 1). The TL ranged from 140 to 450 mm for females and from 100 to 520 mm for males, while the median lengths were 340 and 350 mm TL for females and males, respectively. The LWR parameters were 5.4 × 10*^−^*^7^, 6.2 × 10*^−^*^3^, and 2.4 × 10*^−^*^6^ (k) and 3.37, 2.95, and 3.12 (b), respectively, for females, males, and sex combined. The sex ratio was 1.13:1 (X^2^ = 0.36, *p* = 0.5485) (Table S3).

#### 3.1.10. Cuckoo Ray—*Leucoraja naevus* (Müller and Henle, 1841)

The rarity of the species was supported in this study, given that it was only recorded in South of Sicily, with few scattered samples on the slope (Table 1 and Table 3). No biological data were collected.

#### 3.1.11. Starry Ray—*Raja asterias* Delaroche, 1809

The surveys indicated a wide distribution along the sampling area with a low number of specimens. It also showed a wide depth distribution, even though the species was mainly concentrated on the continental shelf. The starry ray was recorded more frequently in South of Sicily, during spring–summer (4.3%) (Table 1 and Table 2), and autumn (2.5%) (Table 3 and Table 4). The TL ranged from 205 to 590 mm and 220 to 550 mm, while the median length was 320 and 390 mm for females and males, respectively. The LWR parameters were 2.4 × 10^−6^, 4.9 × 10^−6^, and 3.2 × 10^−6^ (k) and 3.14, 3.02, and 3.09 (b), respectively, for females, males, and sex combined. The sex ratio was 1.13:1 (X^2^ = 0.36, *p* = 0.5485) (Table S3).

#### 3.1.12. Blonde Ray—*Raja brachyura* Lafont, 1873

In spring–summer, the blonde ray was caught with high frequency of occurrence (0.9%), abundance indices (shelf: BI = 1.2 ± 0.2 kg/km^2^; DI = 0.3 ± 0.2 N/km^2^), and standing stock (9.1 ± 5.6 tons, 10 ± 6 thousand) in South of Sicily (Table 1 and Table 2). Conversely, in autumn, even if was sampled only in 2006 in both areas, it was caught slightly more frequently in Malta Islands (0.2%) (Table 3 and Table 4). The TL ranged from 310 to 410 mm for females and from 300 to 320 mm for males. The median lengths were 360 and 310 mm TL for females and males, respectively. The sex ratio was 0.85:1 (X^2^ = 0.64, *p* = 0.4237) (Table S3).

#### 3.1.13. Thornback Ray—*Raja clavata* Linnaeus, 1758

**Figure 4:**
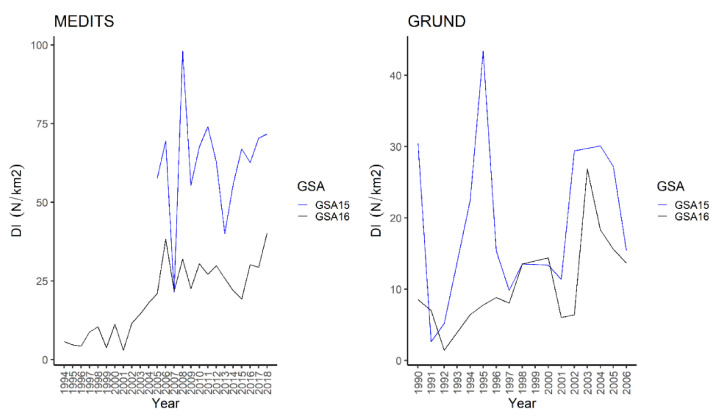
Density Index (DI, N/km^2^) of *Raja clavata* by overall depth in the South of Sicily (GSA16) and Malta Island (GSA15) between 1994 and 2018 during MEDITS–spring summer–(**left**) and between 1990 and 2006 during GRUND–autumn–(**right**).

The species has been recorded across all the considered strata, albeit it seemed more abundant on the shelf (Table 1, Table 2 and Table 3). In spring–summer, the highest frequency of occurrence (53%), abundance (slope: BI = 90.7 ± 15.0 kg/km^2^, DI = 65.1 ± 9.1 N/km^2^), and standing stock (706.8 ± 61.6 tons, 660 ± 49 thousand) were recorded in Malta Island (Table 1 and Table 2). Similarly, in autumn, the species seemed more common and abundant in Malta Island (Table 3 and Table 4). The abundance indices of the thornback ray was positively correlated over the years on the slope during spring-summer in South of Sicily and on the shelf from the other surveys (Table 1, Table 2, Table 3 and Table 4). The yearly DI plot showed that the DI obtained was overall higher from Malta Island during spring-summer; this did not apply to 2007 (Figure 4).

**Figure 5:**
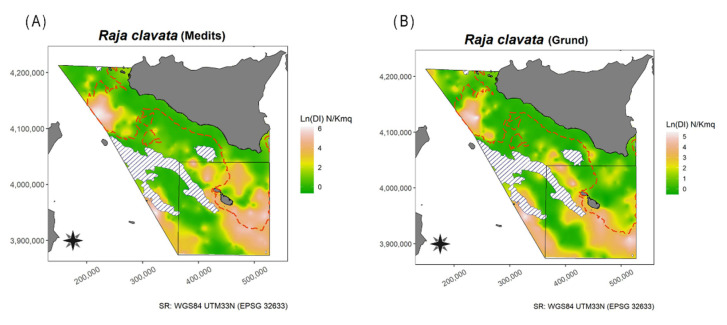
Spatial distribution, in terms of Density Index (DI, N/Km^2^), of *Raja clavata* in South of Sicily (GSA 16) and Malta island (GSA15) during (**A**) MEDITS–spring summer–and (**B**) GRUND–autumn–surveys. The dashed areas denote depths below 800 m not explored in present surveys.

In addition, the Mann–Whitney U test highlighted significant differences for DI (W = 183, *p* < 0.001), BI (W = 194, *p* < 0.001), and frequency of occurrence (W = 196, *p* < 0.001) between areas during spring–summer. The highest values of DI were recorded during autumn in Malta Island, except for 1991, 1998, and 2000; a clear peak was observed in 1995 (Figure 4). In addition, the Mann–Whitney U test showed significant differences between the DI (W = 114, *p* = 0.01449), BI (W = 175, *p* = 0.008642), and frequency of occurrence (W = 144, *p* < 0.001) between areas during autumn. The spatial distribution of the species in South of Sicily was mainly concentrated in two areas, namely Adventure Bank and in the southernmost sector of the GSA16 (NE Lampedusa Island), whereas in Malta Island it seemed to be more widespread, particularly in the SE part (Figure 5).

**Figure 6:**
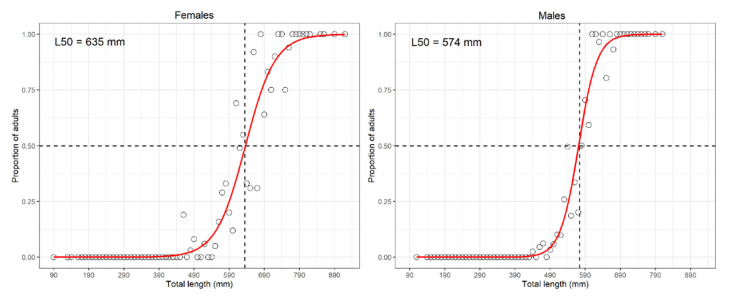
Logistic curves describing the proportion of *Raja clavata* matures by length (Total Length, mm) for females and males; L_50_ denotes the estimated size at first maturity from the MEDITS–spring summer–survey carried out in the South of Sicily (GSA16).

The TL ranged from 95 to 1100 and from 110 to 810 mm TL for females and males, respectively. The LFD was quite comparable between sexes, although the median length was slightly lower for females (480 mm) than males (500 mm). The Kolmogorov–Smirnov test showed no significant statistical differences in the LFDs over the years for females, whilst a significant difference was observed for males only in 1995. The LWR parameters were 6.0 × 10^−7^, 7.6 × 10^−7^, and 6.8 × 10^−7^ (k) and 3.36, 3.33, and 3.35 (b), respectively, for females, males, and sex combined. The ELEFAN growth estimates confirmed a larger size (L∞: 1260 vs. 1100 mm) and lower growth rate (K: 0.16 vs. 0.26) in females than males. The Z/K ratios were 1.32 (and 0.95 (Confidence Interval: 0.94–0.96) for females and males, respectively. The estimated values of L_50_, through the logistic approach, were 635 mm for females and 574 mm for males (Figure 6). The sex ratio was 1:1 (Table S3).

#### 3.1.14. Brown Ray—*Raja miraletus* Linnaeus, 1758

**Figure 7:**
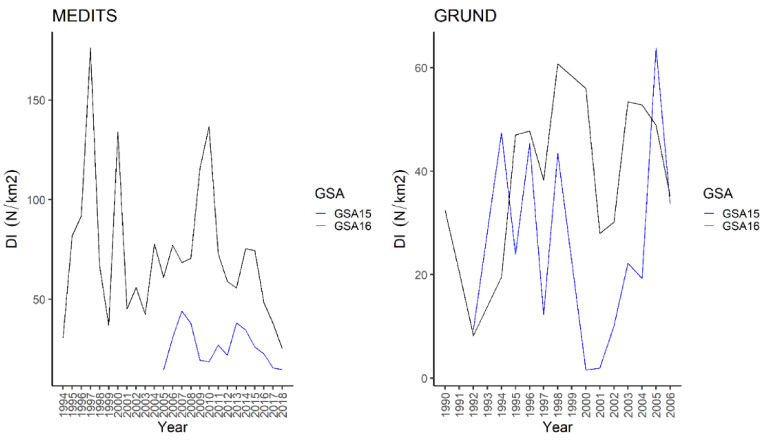
Density Index (DI, N/km^2^) of *Raja miraletus* by overall depth in the South of Sicily (GSA16) and Malta island (GSA15) between 1994 and 2018 during MEDITS–spring summer–(**left**) and between 1990 and 2006 during GRUND–autumn–(**right**).

The species was recorded from 10 to 800 m; however, it was more abundant in the b stratum, 50–100 m, namely, the inner shelf. The brown ray was more frequent in Malta Island (spring–summer = 24%; autumn = 27.6%), although it was more abundantly caught in South of Sicily during spring-summer and autumn (e.g., South of Sicily: spring–summer 371.9 ± 34.2 tons, 2282 ± 224 thousand; autumn 1211 ± 125 thousand) (Table 1, Table 2, Table 3 and Table 4). The abundance indices showed a significant, positive correlation only during autumn in South of Sicily on the shelf (Table 3). The yearly DI plot showed that the DI was always higher in MED16 (Figure 7).

**Figure 8:**
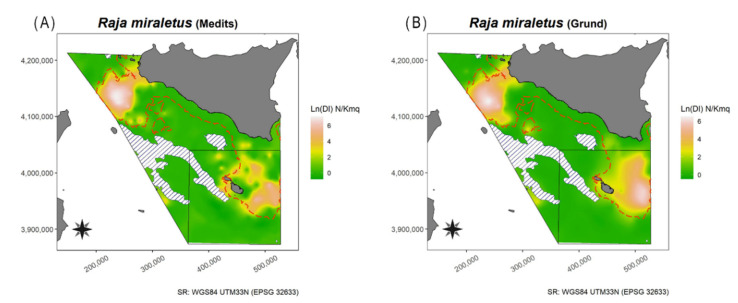
Spatial distribution, in terms of Density Index (DI, N/Km^2^), of *Raja miraletus* in South of Sicily (GSA 16) and Malta island (GSA15) during (**A**) MEDITS–spring summer–and (**B**) GRUND–autumn–surveys. The dashed areas denote depths below 800 m not explored in present surveys.

The Mann–Whitney U test highlighted significant differences in DI (W = 9, *p* < 0.001), BI (W = 31, *p* = 0.001433), and frequency of occurrence (W = 142.5, *p* = 0.04232) between areas in spring summer. As for autumnal survey the DI was generally higher in South of Sicily, except for 1992, 1994, and 2005 (Figure 7). Significant differences were observed for DI (W = 58, *p* = 0.0411) but not for BI (W = 66, *p* = 0.09322) and frequency of occurrence (W = 136.5, *p* = 0.176) between areas in autumn. The map of the spatial distribution showed that the brown ray in South of Sicily was mainly distributed on Adventure Bank, whereas in Malta Island it was mainly concentrated in the NE part. In both areas, the species was more present mainly on the shelf (Figure 8).

**Figure 9:**
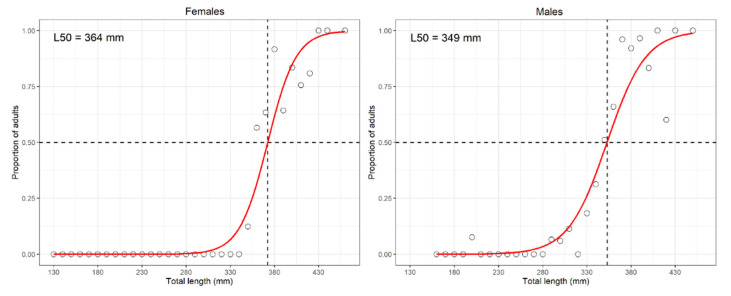
Logistic curves describing the proportion of *Raja miraletus* matures by length (Total Length, mm) for females and males; L_50_ denotes the estimated size at first maturity from the MEDITS–spring summer–survey carried out in the South of Sicily (GSA16).

The TL ranged from 135 to 470 and from 135 to 460 mm for females and males, respectively. The LFD stability showed no significant statistical difference between years for both sexes, while the median length was 320 and 330 mm for females and males, respectively. The LWR parameters were 2.7 × 10^−7^, 4.2 × 10^−7^, and 3.4 × 10^−7^ (steepness) and 3.36, 3.33, and 3.35, respectively, for females, males, and sex combined. ELEFAN resulted in L∞ of 840 and 800 mm TL, while K was 0.36 and 0.41 for females and males, respectively. Differently from other rays, the Z/K value was quite higher than expected for both sexes, that is, 2.96 and 3.13 for females and males, respectively (the corresponding Z would be around 1.2, a value which looks more realistic). The estimated L_50_ values were 364 and 349 mm TL (logistic approach; Figure 9) for females and males, respectively. The sex ratio was 0.96:1 (X^2^ = 0.04, *p* = 0.8415) (Table S3).

#### 3.1.15. Spotted Ray—*Raja montagui* Fowler, 1910

Survey data showed a wide spatial distribution and a preference for coastal and shelf areas (mainly inner shelf), although the species was found up to 500 m deep. In spring-summer, the highest frequency of occurrence (9%), abundance indices (shelf: BI = 15.0 ± 0.6 kg/km^2^, DI = 3.3 ± 1.4 N/km^2^), and standing stock (28.6 ± 8.4 tons; 103 ± 38 thousand) were observed from Malta Island (Table 1 and Table 2). In Malta Island, the BI on the shelf was higher than that on the slope, while the DI was higher on the slope (Table 2). This could be attributed to the bathymetric segregation of the species across ontogeny, i.e., juveniles are more abundant on the slope. In autumn, the spotted ray was more frequently caught in South of Sicily (5.2%), with the highest abundance indices on the shelf (BI = 0.5 ± 0.1 kg/km^2^; DI = 1.8 ± 0.4 N/km^2^) (Table 3 and Table 4). In Malta Island, the abundance indices were positively correlated over the years on the slope in spring–summer (Table 2), whereas in autumn the abundance indices were only positively correlated to the years on the shelf, i.e., 0.50 (Table 4). The TL ranged from 205 to 520 mm for females and from 200 to 495 mm for males, with the median lengths being 325 and 345 mm for females and males, respectively. The LWR parameters were 2.8 × 10^−7^, 2.0 × 10^−7^, and 2.8 × 10^−7^ (k) and 3.51, 3.55, and 3.50 (b, i.e., a strong, positive allometry) for females, males, and sex combined, respectively. The sex ratio was 1.22:1 (X^2^ = 1, df = 1, *p* = 0.3173) (Table S3).

#### 3.1.16. Speckled Ray—*Raja polystigma* Regan, 1923

The speckled ray seemed to inhabit the shelf. In spring–summer, the species was most frequently encountered (1%) and most abundant (BI 0.6 ± 0.4 kg/km^2^; DI 0.8 ± 0.5 N/km^2^) on the slope in the Malta Island (Table 1 and Table 2). In autumn, it was caught only in South of Sicily in 1995; therefore, a very low abundance indices were recorded (Table 3). The TL ranged from 225 to 405 mm for females and from 300 to 475 mm for males. Considering the combined sexes, the b parameter of the LWR showed a strong, positive allometric growth (3.45), while the steepness (k) was 4.4 × 10^−7^. The sex ratio was 0.64:1 (X^2^ = 4.84, df = 1, *p* = 0.02781) (Table S3).

#### 3.1.17. Rough Ray—*Raja radula* Delaroche, 1809

The surveys suggested that the rough ray showed preference towards the shelf (mainly the outer shelf, 100–200 m deep). In spring–summer, the species was frequently encountered in Malta Island (1.7%), particularly on the shelf (BI = 4.8 ± 2.9 kg/km^2^; DI = 5.2 ± 2.7 (N/km^2^) (Table 1 and Table 2). Similarly, in autumn, the highest frequency of occurrence (2.4%) and standing stock (20.5 ± 11.7 tons; 55 ± 36 thousand) were observed from the Malta Island; here the species was found only on the shelf (BI = 1.8 ± 1.0 kg/km^2^; DI = 4.8 ± 3.2 N/km^2^) (Table 3 and Table 4). The abundance of the rough rays was only positively correlated to the shelf during autumn in Malta Island (Table 4). The median lengths were 430 and 445 mm TL, respectively, for females and males. The sex ratio was 0.51:1 (X^2^ = 10.24, *p* = 0.001374) (Table S3).

#### 3.1.18. Undulate Ray—*Raja undulata* Lacepède, 1802

In surveys, it was encountered only in 2004 during autumn survey in Malta Island within the b stratum (51–100 m). Therefore, the resulting abundance indices were very low, i.e., <0.1 (Table 4). No biological data were collected.

#### 3.1.19. White Skate—*Rostroraja alba* (Lacepède, 1803)

The white skate was most frequently encountered on the shelf, but it could reach depths of 800 m. In spring–summer, the highest frequency of occurrence (1.6%), abundance indices (shelf: BI = 3.0 ± 1.1 kg/km^2^, DI = 0.6 ± 0.2 N/km^2^), and standing stock (57.1 ± 15.3 tons, 11 ± 3 thousand) of the white stake were observed from South of Sicily (Table 1 and Table 2). Similarly, in autumn, the species was caught more frequently from South of Sicily (2.5%). The abundance indices of the white skate were positively correlated across years on the shelf only during spring–summer in South of Sicily (Table 1). The TL ranged from 325 to 1450 mm for females and from 315 to 1320 mm for males. The median lengths were 890 and 740 mm (TL), respectively, for females and males. The steepness values of the LWR were 1.9 × 10^−6^, 1.7 × 10^−6^, and 1.7 × 10^−6^ for females, males, and sex combined, and the b parameter had positive allometry for females (3.18), males (3.09), and sex combined (3.14). The sex ratio was 0.75:1 (X^2^ = 1.96, *p* = 0.1615) (Table S3).

#### 3.1.20. Common Stingray—*Dasyatis pastinaca* (Linnaeus, 1758)

The common stingray was more frequent and abundant in Malta Island in both seasons, in comparison to South of Sicily (Table 1, Table 2, Table 3 and Table 4). In spring–summer, a positive correlation for the abundance was observed in South of Sicily on the shelf, while a negative correlation was observed from Malta Island (Table 1 and Table 2). The TL ranged from 315 to 970 mm for females and from 510 to 710 mm for males, respectively. The median lengths were 830, 680, and 710 mm TL for females, males, and for sex combined, respectively. The steepness of the LWR was 3.0 × 10^−4^, whereas the b parameter was 2.48 (a quite extreme negative allometry) for the sexes combined. The sex ratio was 1.94:1 (X^2^ = 10.24, *p* = 0.001374) (Table S3).

#### 3.1.21. Blue Stingray—*Pteroplatytrygon violacea* (Bonaparte, 1832)

The surveys show that the species in South of Sicily was only encountered once in spring-summer (2012) and once in autumn (1996); in both cases, very low values of abundance indices were observed (Table 1 and Table 3). The rarity could be related to the prevalent pelagic behavior of this species; for the surveys, it was classified as incidental. No biological data were collected.

#### 3.1.22. Bull Ray—*Aetomylaeus bovinus* (Geoffroy Saint-Hilaire, 1817)

Few scattered records were recorded during spring–summer in South of Sicily, in one spot next to Pozzallo (southeast Sicilian coast) and along the northeast of Linosa Island. *A. bovinus* has only been recorded in South of Sicily at depths ranging from 10 to 50 m during spring-summer, whereas it was caught between 200 and 500 m in autumn (Table 1 and Table 3); this could be related to the seasonal migration of the species. The frequency of occurrence was 0.4% and 0.1, respectively, during spring-summer and autumn from the South of Sicily. On the shelf, in South of Sicily, DI and BI were 0.2 ± 0.1 N/km^2^ and 2.2 ± 1.6 kg/km^2^, respectively, in spring–summer, while in autumn both abundance indices resulted lower than 0.1. In South of Sicily, the standing stock in weight were 41.7 ± 23.0 and 0.4 ± 0.3 tons, while standing stock in number were 3 ± 2 and 0.5 ± 0.3 thousand, respectively, in spring–summer and autumn. This suggests that it reached large sizes and also indicates a seasonality trend in the catches. No significant correlation for the abundance indices over the years was observed (Table 1 and Table 3). The median lengths were 2650 and 820 mm TL for females and males; the marked differences between sexes may be due to the few sampled specimens. The sex ratio was 0.96:1 (X^2^ = 0.04, *p* = 0.8415) (Table S3).

#### 3.1.23. Common Eagle Ray—*Myliobatis aquila* (Linnaeus, 1758)

The common eagle ray’s preferential stratum was the shelf. It was more frequent and abundant in Malta Island than South of Sicily in both surveys (Table 1, Table 2, Table 3 and Table 4). In spring–summer, a significant, positive correlation was observed between the abundance indices and years from South of Sicily (Table 1). In autumn, a significant, positive correlation between abundance indices and years was only observed from Malta Island (Table 4). The TL ranged from 425 to 1110 mm in females and from 435 to 775 mm in males. The median lengths were 530, 580, and 542 mm TL for females, males, and sex combined, respectively. The steepness was 4 × 10^−9^, and the b parameter of the LWR was 4.01 for the sexes combined (a quite extreme positive allometry, likely reflecting the low representativeness of the present samples). The sex ratio was 0.72:1 (X^2^ = 2.56, *p* = 0.1096) (Table S3).

## 4. Discussion

### 4.1. General Consideration

In the Mediterranean, according to the most recent assessment of the IUCN, 50% of the considered rays (16 of 32 species) are facing a high risk of extinction [11]. In addition, the lack of most basic life-history data and taxonomic uncertainties make it harder to assess their status (see, e.g., in [54,55]). Bradai et al. [29] underlined that some neritic species have almost locally disappeared (e.g., *R. alba*) or should be considered as highly depleted (*R. polystigma*), whereas few species are quite stable (e.g., *R. clavata* and *R. miraletus*), although considered by the authors in a depleted status (i.e., quite low standing stock in weight). In particular, *R. clavata*, *R. radula*, and *R. miraletus* are the species most commonly caught and landed by trawlers [36,56].

In the Strait of Sicily, Giudicelli [57] in the middle of the 70th explored 10 fishing grounds between 70 and 400 m depths and reported a maximum of 23.3 kg/h of “Rays” (this corresponds to around 233 kg/km^2^). Recently, in the GSA16, Geraci et al. [26] found quite stable trends by analyzing the pooled BI and DI of the recorded batoids from 1994 to 2013, and the BI/DI ratio remained heterogeneous.

In the present study, among all the species analysed only three taxa (*R. clavata*, *R. miraletus*, and *D. oxyrinchus*) showed relatively high abundance indices so much that they mightbe considered profitable for fisheries. For some coastal species, e.g., *T. marmorata*, recreational fisheries (i.e., spear fishing) could be an important cause of mortality (Ragonese pers. obs.). Besides, differences in the DI of *D. oxyrinchus*, *R. clavata*, and *R. miraletus* between the studied areas were recognized attributable to the presence of a large protected area, namely the Maltese Economic Exclusive Zone, in Malta Island (GSA15) with limited access to fishing activities (i.e., a limited number of trawlers). Differences may also be due to the great abundance of Fish Aggregating Devices (FADs), which make large portions of the seabed non-trawable as several thousands of heavy stones used as anchors are left at the bottom at the end of the fishing season. This hypothesis could be corroborated by the fact that the largest catches of *R. clavata*, the most landed species, are mainly produced by boats with bottom trawls and set nets, while minimum quantities are caught with long-lines [25]. Considering that from 2009 onwards only MEDITS (spring–summer) was maintained, the re-implementation of a second MEDITS Autumn survey (as the Italian GRUND) could greatly improve the monitoring of these important animals. Overall, the spatial distribution of these taxa highlighted a widespread distribution in Malta Island (GSA15) and a quite unbalanced distribution in the South of Sicily (GSA16), with two main hotspots, i.e., Adventure Bank and the waters close to Linosa Island (Figure 3, Figure 5, and Figure 8). Generally, our results, especially for Adventure Bank and the waters of Linosa Island, are comparable to the spatial analysis reported by previous studies in the area (see, e.g., in [23,26,58,59]). Herein, present findings are summarily compared among species with data reported in the literature, in general for the Mediterranean Sea and in particular for the Strait of Sicily.

#### 4.1.1. Great Torpedo Ray—*Tetronarce nobiliana* (Bonaparte, 1835)

In the North Mediterranean, the highest frequency (9.5%) and abundances were recorded in GSA01 (Northern Alboran Sea; DI = 1 ± 0.8 N/km^2^, BI = 3.5 ± 3.7 kg/km^2^) [27]. In the Strait of Sicily, Lanfranco [60] stated that this species is usually rare or occasionally frequent off the Southern coast of Sicily, and the same information has been reported by other authors [61,62]. Arena and Li Greci [63] reported isolated records off west of the Egadi Islands. In Tunisia, this species was reported in the north [64,65]. The maximum TL was slightly inferior to the other Mediterranean areas. The slope of the LWR was lower when compared with Turkish specimens [66,67] whereas the sex ratio was higher when compared to Basusta et al. [66] and lower than those reported by Kaya and Basusta [67] (Table S3).

#### 4.1.2. Marbled Electric Ray—*Torpedo marmorata* Risso, 1810

The distribution of this species in the Mediterranean does not seem to be homogeneous. In the Adriatic Sea the species seems to be more common than the other two indigenous Torpedo species (*T. torpedo* and *T. nobiliana*) [68], and it is also relatively abundant in the northern Mediterranean [27], compared to other batoid species [69]. In the Strait of Sicily, it has been historically described as common or very common [60,61,62]. This species is caught mainly off the north coast of Tunisia and more rarely in the Gulf of Tunis and the Gulf of Gabes [70]. The maximum TL was bigger when compared to specimens analyzed by Duman and Başusta [71] in Turkey, whereas it was lower when compared to the other areas. Similar slope values of the LWR to Turkish [71,72,73] and Italian [74,75] specimens were found, whereas a quite low value was recorded in Lebanon [76], which may be due to the small sample size. The sex ratio was quite similar to that found by Consalvo et al. [75] and Yeldan and Gundogdu [72], whereas it was inferior when compared to Duman and Basusta [71], Abdel-Aziz [77], and Bellodi et al. [74] (Table S3).

#### 4.1.3. Common Torpedo—*Torpedo torpedo* (Linnaeus, 1758)

This was an infrequent species, recorded with the highest frequency (3.7%) in GSA19 (Western Ionian Sea), while the highest abundances were detected in GSA01 (DI = 3.0 ± 3.5, BI = 1.9 ± 2.1) [27]. The maximum TL recorded in the present study was lower when compared to Tunisian [78] and Italian (regions of central western Italy) specimens, whereas it was bigger than Egyptian specimens. The slope of the LWR was higher and lower when compared, respectively, to male and female specimens from Latium, Italy [75]. The sex ratio was higher than that for Tunisian [78] and Italian specimens [74,75] (Table S3).

#### 4.1.4. Gray Skate—*Dipturus batis* (Linnaeus, 1758)

Off the Strait of Sicily, taxonomic uncertainties make the available information on abundance not fully reliable. Indeed, the low consistency, as well as the doubts concerning historical records, poses questions over the possible misidentification of this species with regard to its congeneric species, *D. nidarosiensis*. The maximum TL recorded during surveys was lower than those reported by Serena et al. [79,80] (Table S3).

#### 4.1.5. Norwegian Skate—*Dipturus nidarosiensis* (Storm, 1881)

Very little information about the Mediterranean abundance of the species is available. In GSA11 (Sardinia), 14 specimens were caught between 2005 and 2008 [81]. More recently, *D. nidarosiensis* was found also in the Ionian Sea and the Adriatic Sea [82,83,84], as well as in the Alboran Sea where eight specimens were caught between 2013 and 2016 [85]. Recently, Carugati et al. [86] provided new evidence of the occurrence of *D. nidarosiensis* in the Central-Western Mediterranean Sea and stated the lack of Atlantic-Mediterranean genetic divergence.

#### 4.1.6. Longnosed Skate—*Dipturus oxyrinchus* (Linnaeus, 1758)

This species was recorded in all areas covered by the MEDITS survey, with exception of GSA06 (Northern Spain) and 17 (Northern Adriatic Sea). The highest values of frequency, DI, and BI were recorded in GSA08 (Corsica), respectively, 82.7%, 26 ± 1 N/km^2^, and 24.4 ± 5.7 kg/km^2^ [27]. Off the South Coasts of Sicily, this species is reported as ubiquitarian [22]. In the present study, *D. oxyrinchus* was more abundant close to Linosa Island, NW and SE Malta Island, in deeper water (Figure 3). Concerning the biological aspects of *D. oxyrinchus*, the maximum size appears to be smaller than that reported by Serena et al. [79]; however, this is similar to other Mediterranean areas [31,87]. The positive allometry indicated by the b parameter of the LWR, estimated in the present study, was also comparable to other areas [73,88,89]. On the contrary, the L_50_ was higher and lower when compared, respectively, to male and female specimens from the Gulf of Gabes [31], North Aegean Sea [88] and Syrian waters [89], while it was lower when compared to those from Sardinian waters [87]. Strangely, the L_50_ estimates, resulted lower for females than males; this probably maybe due to low representativity of the data about males.The sex ratio was quite unbalanced towards females, as reported in Syrian waters [89], whereas, in other areas, it was quite balanced (e.g., Tunisia [31]; North Aegean Sea [88]). Estimates of L∞ in the the South of Sicily were quite similar to the other Mediterranean areas, with the exception of the Aegean Sea [88] (Table S3). On the other hand, the calculated K was higher. From these estimates, it is possible to observe that stocks might be affected by fishing activities in a similar manner to natural, undisturbed conditions. The shape of the LFD indicates a Z/K value less than or around 1, i.e., K-oriented life history traits [44,90].

#### 4.1.7. Sandy Ray—*Leucoraja circularis* (Couch, 1838)

In the North Mediterranean, higher frequencies were recorded in GSA11 (8.6) and GSA28 (Marmara Sea; 8.3%), while the highest abundance was recorded in GSA05 (Bale-aric Islands; DI = 2 ± 3 N/km^2^, BI = 2.1 ± 2.6 kg/km^2^) ([27]). Off the South coasts of Sicily, the species was more prevalent in the southeastern area, but it was also caught in the western area [22]. The maximum TL recorded during surveys (F: 880, M: 750 mm TL) was lower than that reported by Serena [79] and Ebert & Stehmann [91] but resulted quite similar to the other Mediterranean areas [92,93] (Table S3).

#### 4.1.8. Shagreen Ray—*Leucoraja fullonica* (Linnaeus, 1758)

Follesa et al. [27], based on the MEDITS data from 2012 to 2015, found that the species was only present in GSA25 (Cyprus).

#### 4.1.9. Maltese Ray—*Leucoraja melitensis* (Clark, 1926)

This species seems to be very rare. Indeed, Follesa et al. [27] recorded its presence only in GSA16 and GSA22 (Aegean Sea). The maximum TL of the present study was slightly higher compared to the Mediterranean studies (420 mm TL [22]; 500 mm TL [79,94]. In the present study, the parameters of the LWR were for the first time estimated in the Mediterranean. The slope (C: 3.12) was quite similar to that estimated globally by Froese et al. [95], i.e., (C: 3.22).

#### 4.1.10. Cuckoo Ray—*Leucoraja naevus* (Müller and Henle, 1841)

In the North Mediterranean, this species was found with the highest frequency and BI (25.7 ± 11.7 kg/km^2^) in GSA05, while the highest DI (26.0 ± 29.0 N/km^2^) was recorded in GSA01 [27].

#### 4.1.11. Starry Ray—*Raja asterias* Delaroche, 1809

This is a frequently found species, recorded in almost all the areas covered by MEDITS, with the exception of GSA20 (Eastern Ionian Sea), GSA23 (Crete), and GSA25 [27]. Abella et al. [24] analyzed MEDITS data from 1994 to 2013 and found the highest DI (6.4 N/km^2^) and BI (5.3 kg/km^2^) in GSA11, followed by GSA16 (DI = 3 N/km^2^). The highest average individual weight (g) was reported, in decreasing order, for GSA 11 and GSA10 (Southern and Central Tyrrhenian Sea) as well as GSA19 and GSA09 (Ligurian Sea and Northern Tyrrhenian Sea). Ferrà et al. [54], analyzed the data obtained from a modified beam trawl in GSA17 (the so-called “rapido” survey, i.e., Solemon) held between 2005 and 2014, and they found an increasing trend in the occurrence of this species over the years. The maximum TL was lower when compared to other Mediterranean specimens [79,96]. The slopes of the LWR were similar to those reported in the Mediterranean [97,98,99] except for the GSA09 (Ligurian & North Tyrrhenian Sea, [100]). The sex ratio was quite balanced between sexes as reported in other areas [101,102] (Table S3).

#### 4.1.12. Blonde Ray—*Raja brachyura* Lafont, 1873

In the North Mediterranean, this species was the most frequent (18.3%) and abundant (DI = 41.0 ± 38.0 N/km^2^, BI = 26.0 ± 28.6 kg/km^2^) in GSA11 [27]. In the Strait of Sicily, it appears to be relatively rare [22,102,103,104], even though it is not known whether this species was always this rare. Little is known about the abundance of this species along the northwest African coastlines; however, Serena et al. [69] reported its presence from Algeria to Egypt. The maximum TL was lower compared to Mediterranean specimens. Similarly to the present study, Catalano et al. [104] found the sex ratio slightly unbalanced towards males, whereas Porcu et al. [105] reported a sex ratio skewed towards females (Table S3).

#### 4.1.13. Thornback Ray—*Raja clavata* Linnaeus, 1758

This species was recorded across all the North Mediterranean, with exception of GSA19 [27]. On the basis of data collected in Italian seas during 1994–2013 MEDITS, this species was more abundant in Sardinia waters (BI = 27.2 kg/km^2^, DI = 40.5 N/km^2^) and in South of Sicily (BI = 17 kg/km^2^, DI = 17 N/km^2^). The highest mean weight values were reported for Northern Adriatic Sea followed by South of Sicily [25]. Surveys conducted on the Italian and African sides of the Strait of Sicily between 1985 and 2002 revealed negative abundance trends in the past, although Garofalo et al. [58] reported signs of recovery. The abundance on the Italian side of the Strait of Sicily between 1997 and 2004 was approximately five times lower than the abundance on the African side; this was based on higher historical fishing effort on Sicilian than on N African fishing grounds [106]. In the present study, the spatial distribution of *R. clavata* confirms it is a more eurybathic species. Indeed, in the GSA16 it was recorded in Adventure Bank as well as off Linosa Island, whereas in the GSA15 it seems more widespread, particularly concentrated in the SE part of Malta Island (Figure 5). The higher abundances on the edge of GSA16 seem to confirm the analysis performed by Bottari et al. [106] suggesting low connectivity among the stocks occurring there. The TL range of *R. clavata* falls within those reported in the Mediterranean (see, e.g., in [21,32]). The b parameter of the LWR does not differ significantly from those reported by other Mediterranean studies, except for the Balearic Island specimens [107]. Generally, the L_50_ was lower compared to other studies [32,108,109,110,111], except for the Adriatic Sea population which was very similar to the South of Sicily. The regular distribution of the proportions of specimens classified as mature around the logistics indicates a good resolution capability of the used maturity scale and the suitability of the 3a stage as discriminant between immature/mature specimens. In the GSA16, the sex ratio was quite balanced, as was previously reported in the same area [21,66] as well as in Turkey. Conversely, in the Adriatic Sea the sex ratio was slightly unbalanced [112]. The estimates of L∞ confirmed that female specimens are larger than males. The estimates of the L∞ in the South of Sicily were higher when compared to the Adriatic Sea [112], whereas they were similar to the Tunisian area [32] as well as to a previous study carried out in the South of Sicily through vertebrae reading [21]. In the present study, the estimates of the K parameter were higher for both sexes (especially females) than Cannizzaro et al. [21] (Table S3). The Z/K ratios were estimated at 1.32 and 0.95 for females and males, respectively. The values, closer to the expected (undisturbed) value of 1, seem to suggest a high resistance of *R. clavata* (especially in males) to fishing compared to the other batoids; being smaller than females, it is more likely that males are returned to the sea after the sorting operation onboard of fishing vessels and, thus, may survive. Last, the Z/K values are in agreement with the LFD shape, whereas the K estimates are too low; the corresponding Z values (i.e., 0.21 and 0.25) are in agreement with the Z estimates in Abella et al. [25] for South of Sicily (0.25) and Northern Adriatic Sea (0.29). Considering the homogenity of the Z estimation, it is reasonable that the F estimation is also similar. Consequently, given that Abella et al. [25] estimated F_current_ = 0.09 and F_0.1_ = 0.11, the state of *R. clavata* stock is not so bad.

#### 4.1.14. Brown Ray—*Raja miraletus* Linnaeus, 1758

This is a common species, recorded across all the Mediterranean except for Northern Alborean Sea (GSA01) and Gulf of Lion (GSA07) [27]. In the Strait of Sicily, the biomass has fluctuated over the years; however, it remains relatively stable. An overall increase in biomass of 55.6% was reported by Garofalo et al. [58] between 1986 and 2002. However, overall levels of biomass in the northern part of the Strait of Sicily were lower than those off Libya, owing to intensive fishing activities carried out since the 1970s. The overall increase in abundance of brown rays in this area is, therefore, in accordance with the decreasing trend in fishing efforts observed at the beginning of the 21st century due to the displacement of many large trawlers [58]. In the present study, the spatial distribution of *R. miraletus* showed that it is a shallower species, in fact in GSA16 it is mainly present in Adventure Bank and in the NE part of the GSA15 (Figure 8). The size range of *R. miraletus* sampled in the GSA16 was smaller than in other Mediterranean areas. The slope of the LWR was similar to the estimates from the Gulf of Gabes [33] and the Adriatic Sea [113], but they were different from those from the Aegean Sea [73], which reported high values for this parameter. The value was also different from that for Lebanese samples, in which an anomalous, negative allometric growth was estimated [76]. In the present study, the L_50_ estimates of the male specimens were similar to those in other Mediterranean areas, whilst the L_50_ from female specimens was smaller [108,114]. Moreover, for *R. miraletus*, the logistic model fitted well the data (proportion of mature specimens) therefore the L_50_ was quite reliable. The sex ratio, as already noted for *R. clavata*, was well balanced. In the GSA16 the estimates of L∞ were higher when compared to the Tunisian waters [30] and were lower than those in Egyptian specimens [114]). The K parameter was higher, suggesting a faster growth rate compared to other areas [30,114] (Table S3). The obtained Z/K value was quite high for both sexes (~3); this could be due to fishing pressure, which could affect the stock. The early sexual maturity of the males, when compared to females, might explain both the lower L∞ and the higher K observed. In fact, the shape of the Z/K plot for males suggested a discontinuity in the growth pattern with a reduction in the growth rate after sexual maturity.

#### 4.1.15. Spotted Ray—*Raja montagui* Fowler, 1910

In the North Mediterranean, this species appears most frequently (25.0%) in Crete (GSA23), while it seems more abundant (D = 17.0 N/km^2^, B = 11.1 kg/km^2^) in the Agean sea (GSA22) [27]. In the Strait of Sicily, it appears to be ubiquitarian, with concentrations in the south and east of Malta, and between Egadi and Pantelleria [22]. The maximum TL was lower than that reported in the Mediterranean [80] (Table S3). The slope of the LWR was never estimated in the Mediterranean, whereas globally a positive allometry was recorded (C: 3.23 [95]).

#### 4.1.16. Speckled Ray—*Raja polystigma* Regan, 1923

In the North Mediterranean, this species was most frequently caught in Cyprus (GSA25), while it was most abundant in Balearic Island (GSA05) (DI = 13.0 ± 8.0 N/km^2^, BI = 37.9 ± 35.6 kg/km^2^) [27]. The maximum TL was lower compared to other Mediterranean studies [79,115,116]. The parameters of the LWR were for the first time presented in the Mediterranean, but the slope of the present study when compared to the global estimate (C: 3.27, [95]) was higher. The sex ratio (0.64:1) was quite unbalanced towards males, whereas in Sardinia it was quite balanced [115,116] (Table S3).

#### 4.1.17. Rough Ray—*Raja radula* Delaroche, 1809

In the North Mediterranean, the species was rarely caught and was in low abundance [27]. Off the Strait of Sicily, limited information is available on the presence and abundance of this species [22,28]. The sex ratio in the present study was very unbalanced, which may be due to the small sample size, whereas in other areas it seems more balanced [34,117,118] (Table S3).

#### 4.1.18. Undulate Ray—*Raja undulata* Lacepède, 1802

This is a very rare species; its occurrence was recorded in the North Mediterranean from 2012 to 2015 only in Agean Sea (GSA22) and Crete (GSA23) [27]. In the Strait of Sicily, the first specimen of the species was reported by Bini [68].

#### 4.1.19. White Skate—*Rostroraja alba* (Lacepède, 1803)

In the Mediterranean, this species is rare; it was recorded most frequently in Corsica (7.1%) and South of Sicily (4.4%), and more abundantly in South od Sicily (DI = 0.7 ± 0.8 N/km^2^) and Balearic Islands (BI = 5.5 ± 11 kg/km^2^) [27]. *R. alba* was not reported in the list compiled by Bombace and Sarà [119] and Arena and Li Greci [63] for the Strait of Sicily. The maximum TL in the present study was inferior compared to that sampled in Tunisia [35]). The *b* parameter of the LWR was inferior when compared to other estimates [35,120]). The sex ratio was very similar to that found by Kadri et al. [35] (i.e., Tunisia: 0.79:1 vs. present study: 0.89:1) (Table S3).

#### 4.1.20. Common Stingray—*Dasyatis pastinaca* (Linnaeus, 1758)

This species has a low frequency of occurrence in all areas covered by the MEDITS survey [69]. In Turkey, the reported frequency of occurrence was 56.0%. The overall mean DI and BI were 55.3 (N/km^2^) and 107.5 (kg/km^2^), respectively [121]. In the Strait of Sicily, historical information on the presence and abundance of this species was reported by Schembri et al. [28]. Frequency in the northern Mediterranean ranged from a minimum of 0.3% (Spain) to 51.8% (Cyprus), while the highest DI (44.0 ± 20.0 N/km^2^) and BI (23.3 ± 9.6 kg/km^2^) were recorded in Sardinia [27]. The maximum TL was very similar to that found by Yeldan & Gundogdu [72], higher compared to Girgin & Basusta [122], and lower compared to that reported by Serena [123], Yeldan et al. [124] and Yigin & Ismen [125]. The slope of the LWR showed a negative allometry, as reported by Filiz & Bilge [73], whereas other studies in the Aegean Sea reported a positive allometry [66,72,121,122,125,126]. The sex ratio was higher compared to the specimens from the Aegean Sea [66,72,122,124,125,126] and Tunisia [127] (Table S3).

#### 4.1.21. Blue Stingray—*Pteroplatytrygon violacea* (Bonaparte, 1832)

Very low frequencies have been recorded in the Mediterranean, with the highest values (2.7%) being observed from Northern Alboran Sea [27].

#### 4.1.22. Bull Ray—*Aetomylaeus bovinus* (Geoffroy Saint-Hilaire, 1817)

From the MEDITS (2012–2015), the species was recorded only in South of Sicily and in Western Ionian Sea (GSA19), with frequency of 1.0% and 4.6%, respectively. The highest DI was observed in GSA19 (1 ± 0.7 N/km^2^), while the highest BI was observed in GSA16 (2.3 ± 4.6 kg/km^2^) [27]. The sex ratio (0.96:1) was very similar to that estimated in Turkey (1:1) by Başusta & Aslan [128].

#### 4.1.23. Common Eagle Ray—*Myliobatis aquila* (Linnaeus, 1758)

In the Mediterranean Sea, the highest frequency (7.1%) was recorded in Crete, while the highest DI (14 ± 14.4 N/km^2^) and BI (42.5 ± 49.6 kg/km^2^) were observed in Northern Adriatic Sea [27]. The maximum TL was lower than that reported in the Mediterranean (C: 2600 mm; [123]). The *b* parameter of the LWR for the sexes combined was higher compared to the estimate provided by Filiz & Bilge [73].

## 5. Conclusions

Despite the general consensus on the bad exploitation status of almost all Mediterranean batoid stocks, the abundance indices of most of them from the Strait of Sicily showed stable or even increasing trends depicting signs of slight recovery. However, few species seem to be in an ongoing depletion (e.g., *D. pastinaca*, *R. radula*, *L. circularis*, and *R. asterias*) although with different orders of magnitude. This pattern may be owed to the combination of, on one hand, reduced fishing capacity and efforts of the South of Sicily fleet, and, on the other, to the presence of the Maltese Economic Exclusive Zone in Malta Island as well as to the great abundance of abandoned Fishing Aggregating Devices which act as dissuaders to trawling and providing further shelters.

A true rebuilding (i.e., local abundances approaching the level corresponding to maximum sustainable yield) could, however, require more time, as demonstrated by the Z/K parameter estimates. The picture provided by the present study indicates the magnitude of the combined effect of fisheries and natural mortality on this vulnerable taxonomic group. However, as reported by Schwamborn [129], some biases may occur in the estimation of Z/K ratio due to (i) the perfect linearization of the data, (ii) the extremely narrow confidence intervals, and (iii) to a possible artifact, i.e., the spurious autocorrelation between cutofflengths and mean lengths. Furthermore, lower values of L_50_ in the GSA16 were observed when compared to other Mediterranean studies for all species, except for male of *D. oxyrinchus* which are likely biased because of the low number of mature specimens. This trend could be an adaptive response of this taxon to the high fishing effort in the area [130]. In conclusion, the lack of detailed quantitative previous information on the batoid of South of Sicily and Malta Island does not allow to judge analytically the current status of the stocks, although the higher abundance of some species within Malta raises some concern for the Sicilian counterpart.

In spite of this relevant historical limitation, at a precautionary level, it seems wise to adopt measures finalised at the protection of these species such as (i) maintenance or a reduction of fishing effort, (ii) improvement of the selectivity of trawl gears (implementation of an ad-hoc modified Turtle Excluder Device) [131,132], (iii) to improve education on responsible fishing by maximizing the number of cartilaginous fish returned to the sea alive (see, e.g., in [133]), (iv) increase collaboration among enterprises and, in general, among stakeholders to define innovative technical solutions (see, e.g., in [53,134,135,136]) and, (v) exploring the feasibility of creating ground aquaculture facilities to raise the spawners, and then taking the ovigerous capsules and implanting them on artificial substrates.

## Figures and Tables

**Table 1:** Synoptic table of batoid abundance sampled during the MEDITS survey (spring–summer) in the GSA16 (South of Sicily), from 1994 to 2018. Percent frequency of occurrence for number of surveys (s%) and positive hauls (f%). Standing Stock (SS) expressed in weight (SSw, tons) and number (SSn, thousand). Stratum distribution and mean biomass index kg/km^2^ (BI) expressed by depth of the macrostratum (10–200 m, shelf; 201–800 m, slope; 10–800 m, overall), mean density index N/km^2^ (DI) by depth of the macrostratum, and Spearman coefficients for BI and DI by depth of the macrostratum.

Species	Frequency of Occurrence		SSw (t) and SSn (Mean ± SE)		Stratum Distribution (Depth Interval in m)					BI (kg/km^2^) Mean ± SE			Spearman Coeff. BI		DI (N/km^2^) Mean ± SE			Spearman Coeff. DI	
	%N. Survey	% Positive Hauls	Tons	Number (Thousand)	10–50	50–100	100–200	200–500	500–800	Shelf	Slope	Overall	Shelf	Slope	Shelf	Slope	Overall	Shelf	Slope
*Tetronarce nobiliana*	76	2.1	12.5 ± 6.7	9 ± 2		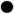	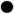	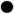	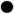	<0.1	0.7 ± 0.4	0.4 ± 0.2	0.05	0.20	0.2 ± 0.1	0.3 ± 0.1	0.3 ± 0.1	−0.03	−0.10
*Torpedo marmorata*	100	8.7	20.8 ± 2.5	69 ± 7	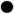	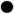	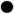	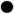	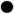	1.2 ± 0.1	0.2 ± 0.0	0.7 ± 0.1	−0.22	−0.17	3.6 ± 0.4	1.0 ± 0.2	2.2 ± 0.2	−0.29	0.08
*Torpedo torpedo*	40	0.8	5.8 ± 3.2	9 ± 3	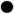		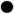	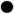		0.1 ± 0.0	0.3 ± 0.2	0.2 ± 0.1	0.49	−0.09	0.6 ± 0.2	<0.1	0.3 ± 0.1	0.49	−0.09
*Dipturus batis*	4	0.1	2.0 ± 2.0	0.2 ± 0.2					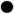	//	0.1 ± 0.1	<0.1	//	NC	//	<0.1	<0.1	//	NC
***Dipturus oxyrinchus***	**100**	**10.6**	**92.3 ± 9.4**	**73 ± 7**			** 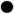 **	** 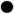 **	** 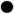 **	**<0.1**	**5.5 ± 0.6**	**2.9 ± 0.3**	**NC**	0.54 *	**<0.1**	**4.3 ± 0.4**	**2.3 ± 0.2**	**NC**	**0.36**
*Leucoraja circularis*	40	0.6	2.6 ± 1.0	2 ± 1			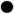	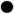	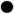	<0.1	0.2 ± 0.1	0.1 ± 0.0	NC	0.37	<0.1	0.1 ± 0.0	0.1 ± 0.0	NC	0.38
*Leucoraja fullonica*	4	0.1	0.5 ± 0.5	0.1 ± 0.1				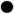		//	<0.1	<0.1	//	NC	//	<0.1	<0.1	//	NC
*Leucoraja melitensis*	96	6.9	26.7 ± 3.0	132 ± 15	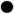	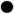	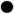	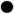	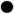	0.4 ± 0.1	1.2 ± 0.2	0.8 ± 0.1	0.54 *	0.04	1.6 ± 0.5	6.5 ± 0.8	4.2 ± 0.5	0.54 *	0.03
*Leucoraja naevus*	4	0.2	0.1 ± 0.1	0.4 ± 0.4				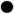		//	<0.1	<0.1	//	NC	//	<0.1	<0.1	//	NC
*Raja asterias*	96	4.3	32.7 ± 4.7	71 ± 12	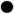	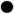	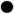	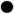	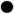	2.1 ± 0.3	0.1 ± 0.1	1.0 ± 0.1	−0.37	0.24	4.4 ± 0.6	0.4 ± 0.3	2.3 ± 0.4	−0.29	0.24
*Raja brachyura*	28	0.9	9.1 ± 5.6	10 ± 6	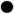	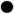	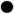			0.3 ± 0.2	//	0.1 ± 0.1	0.41	//	0.3 ± 0.2	//	0.1 ± 0.1	0.42	//
***Raja clavata***	**100**	**19.8**	**603.6 ± 66.4**	**609 ± 70**	** 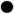 **	** 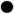 **	** 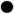 **	** 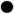 **	** 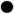 **	**33.3 ± 3.8**	**7.2 ± 1.0**	**19.2 ± 2.1**	0.86 *	0.59 *	**31.8 ± 3.6**	**8.8 ± 1.4**	**19.4 ± 2.2**	0.85 *	0.66 *
***Raja miraletus***	**100**	**18.9**	**371.9 ± 34.2**	**2282 ± 224**	** 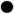 **	** 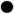 **	** 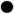 **	** 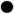 **	** 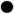 **	**25.4 ± 2.4**	**0.2 ± 0.1**	**11.8 ± 1.1**	**−0.00**	**0.46**	**156.2 ± 15.5**	**1.9 ± 0.4**	**35.2 ± 3.7**	**−0.21**	**0.42**
*Raja montagui*	96	6.0	19.8 ± 2.6	73 ± 13	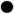	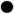	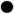	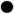		1.3 ± 0.2	0.1 ± 0.0	0.6 ± 0.1	−0.09	0.07	4.7 ± 0.9	0.3 ± 0.1	2.3 ± 0.4	−0.16	0.08
*Raja polystigma*	28	0.5	1.2 ± 0.5	4 ± 1	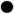	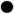		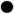		0.1 ± 0.0	<0.1	<0.1	0.45	0.49	0.2 ± 0.1	<0.1	0.1 ± 0.0	0.38	0.49
*Raja radula*	12	0.4	1.2 ± 0.9	1 ± 1	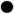					0.1 ± 0.1	//	<0.1	0.07	//	0.1 ± 0.1	//	<0.1	0.06	//
*Rostroraja alba*	64	1.6	57.1 ± 15.3	11 ± 3		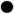		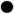	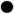	3.0 ± 1.1	0.8 ± 0.3	1.8 ± 0.5	0.73 *	0.13	0.6 ± 0.2	0.1 ± 0.0	0.3 ± 0.1	0.69 *	0.06
*Dasyatis pastinaca*	64	1.2	51.7 ± 13.0	11 ± 2		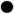	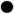	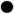		3.6 ± 0.8	<0.1	1.6 ± 0.4	0.73 *	NC	0.8 ± 0.2	<0.1	0.4 ± 0.1	0.67 *	NC
*Pteroplatytrygon violacea*	4	0.1	1.9 ± 1.9	0.2 ± 0.2			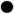			0.1 ± 0.1	//	<0.1	NC	//	<0.1	//	<0.1	NC	//
*Aetomylaeus bovinus*	12	0.4	41.7 ± 23.0	3 ± 2	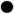					2.2 ± 1.6	//	1.0 ± 0.7	0.01	//	0.2 ± 0.1	//	0.1 ± 0.1	−0.02	//
*Myliobatis aquila*	44	0.7	31.0 ± 9.7	8 ± 3	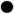	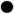				1.0 ± 0.3	//	0.5 ± 0.1	0.71 *	//	0.6 ± 0.2	//	0.3 ± 0.1	0.73 *	//

Significant correlations are noted by an asterisk (Spearman coefficients > ±0.50); NC: Not Considered because of the low sample size; //: Not Available. In bold, the species is above the fixed thresholds (f% ≥ 10; SSw ≥ 50 tons at least in two surveys). Black dots indicate the depth range within which the species was sampled.

**Table 2:** Synoptic table of batoid abundance sampled during the MEDITS survey (spring–summer) in the GSA15 (Malta Island), from 2005 to 2018. Percent frequency of occurrence for number of surveys (s%) and positive hauls (f%). Standing Stock (SS) expressed in weight (SSw, tons) and number (SSn, thousand). Stratum distribution and mean biomass index kg/km^2^ (BI) expressed by depth of the macrostratum (10–200 m, shelf; 201–800 m, slope; 10–800 m, overall), mean density index N/km^2^ (DI) by depth of the macrostratum, and Spearman coefficients for BI and DI by depth of the macrostratum.

Species	Frequency of Occurrence		SSw (t) and SSn (Mean ± SE)		Stratum Distribution (Depth Interval in m)				BI (kg/km^2^) Mean ± SE			Spearman Coeff. BI		DI (N/km^2^) Mean ± SE			Spearman Coeff. DI	
	% N. Survey	% Positive Hauls	Tons	Number (Thousand)	50–100	100–200	200–500	500–800	Shelf	Slope	Overall	Shelf	Slope	Shelf	Slope	Overall	Shelf	Slope
*Tetronarce nobiliana*	29	1.4	1.5 ± 1.1	3 ± 1		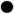	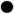		<0.1	0.2 ± 0.2	0.1 ± 0.1	−0.38	−0.48	0.1 ± 0.1	0.4 ± 0.2	0.3 ± 0.1	−0.38	−0.49
*Torpedo marmorata*	100	11.1	9.2 ± 2.2	35 ± 7	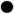	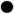	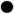		1.8 ± 0.5	0.1 ± 0.0	0.9 ± 0.2	−0.33	0.11	6.5 ± 1.3	0.7 ± 0.3	3.3 ± 0.6	−0.16	0.05
***Dipturus oxyrincus***	**100**	**30.94**	**262.4 ± 26.5**	**202 ± 36.8**		** 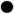 **	** 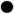 **	** 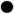 **	**5.1 ± 1.1**	**40.5 ± 5.4**	**24.8 ± 2.5**	**0.15**	**−0.15**	**2.5 ± 0.5**	**32.3 ± 4.8**	**19.0 ± 2.9**	0.53 *	**−0.34**
*Leucoraja circularis*	79	5.7	247.5 ± 233.8	20 ± 7		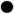	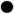	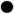	0.3 ± 0.3	41.8 ± 39.8	23.4 ± 22.1	−0.49	−0.27	0.2 ± 0.2	3.3 ± 1.2	1.9 ± 0.7	−0.49	−0.09
*Leucoraja fullonica*	43	1.8	2.1 ± 1.5	3 ± 1			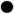	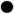	//	0.4 ± 0.2	0.2 ± 0.1	//	−0.19	//	0.5 ± 0.2	0.3 ± 0.1	//	−0.12
*Leucoraja melitensis*	93	10.5	20.9 ± 3.8	105 ± 19	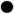	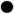	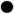	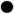	0.3 ± 0.1	3.3 ± 0.6	2.0 ± 0.4	0.05	0.34	1.9 ± 1.0	16.3 ± 2.9	9.9 ± 1.8	0.04	0.24
*Raja asterias*	7	0.2	<0.1	1 ± 1			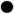		//	<0.1	<0.1	//	NC	//	<0.1	<0.1	//	NC
*Raja brachyura*	14	0.3	2.2 ± 1.8	1 ± 1		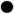	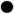		0.4 ± 0.4	<0.1	0.2 ± 0.2	NC	NC	0.2 ± 0.2	<0.1	0.1 ± 0.1	NC	NC
***Raja clavata***	**100**	**52.8**	**706.8 ± 61.6**	**660 ± 49**	** 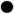 **	** 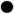 **	** 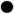 **	** 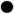 **	**90.7 ± 15.0**	**47.7 ± 5.2**	**66.8 ± 5.8**	0.80 *	**−0.44**	**59.0 ± 7.6**	**65.1 ± 9.1**	**62.4 ± 4.7**	0.74 *	**−0.41**
***Raja miraletus***	**100**	**24.3**	**70.3 ± 8.0**	**277 ± 27**	** 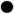 **	** 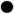 **	** 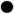 **	** 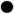 **	**14.1 ± 1.7**	**0.7 ± 0.3**	**6.6 ± 0.8**	**0.26**	**−0.29**	**53.8 ± 5.4**	**4.0 ± 2.0**	**26.2 ± 2.6**	**−0.13**	**−0.23**
*Raja montagui*	93	9.4	28.6 ± 8.4	103 ± 38	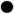	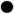	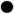	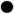	1.5 ± 0.6	3.6 ± 1.1	2.7 ± 0.8	−0.02	0.83 *	3.3 ± 1.4	14.9 ± 5.8	9.7 ± 3.6	0.03	0.83 *
*Raja polystigma*	29	1.3	4.3 ± 2.6	5 ± 3	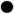		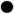		0.1 ± 0.1	0.6 ± 0.4	0.4 ± 0.2	NC	−0.41	<0.1	0.8 ± 0.5	0.5 ± 0.3	NC	−0.44
*Raja radula*	50	1.7	22.4 ± 13.6	24 ± 13	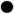				4.8 ± 2.9	//	2.1 ± 1.3	−0.42	//	5.2 ± 2.7	//	2.3 ± 1.2	−0.42	//
*Rostroraja alba*	7	0.3	0.4 ± 0.4	4 ± 4			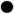		//	<0.1	<0.1	//	NC	<0.1	0.7 ± 0.7	0.4 ± 0.4	//	NC
*Dasyatis pastinaca*	71	4.3	60.2 ± 17.6	24 ± 6	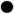	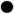		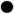	12.8 ± 3.7	<0.1	5.7 ± 1.7	−0.97 *	NC	5.1 ± 1.4	<0.1	2.3 ± 0.6	−0.78 *	NC
*Myliobatis aquila*	64	4.6	56.5 ± 16.8	23 ± 7	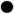	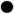			12.0 ± 3.6	//	5.3 ± 1.6	−0.29	//	4.8 ± 1.5	//	2.1 ± 0.7	−0.40	//

Significant correlations are noted by an asterisk (Spearman coefficients > ±0.50); NC: Not Considered because of the low sample size; //: Not Available. In bold, the species is above the fixed thresholds (f% ≥ 10; SSw ≥ 50 tons at least in two surveys). Black dots indicate the depth range within which the species was sampled.

**Table 3:** Synoptic table of batoid abundance sampled during the GRUND survey (autumn) in the GSA16 (South of Sicily), from 1990 to 2006. Percent frequency of occurrence for number of surveys (s%) and positive hauls (f%). Standing Stock (SS) expressed in weight (SSw, tons) and number (SSn, thousand). Stratum distribution and mean biomass index kg/km^2^ (BI) expressed by depth of the macrostratum (10–200 m, shelf; 201–800 m, slope; 10–800 m, overall), mean density index N/km^2^ (DI) by depth of the macrostratum, and Spearman coefficients for BI and DI by depth of the macrostratum.

Species	Frequency of Occurrence		SSw (t) and SSn (Mean ± SE)		Stratum Distribution (Depth Interval in m)					BI (kg/km^2^) Mean ± SE			Spearman Coeff. BI		DI (N/km^2^) Mean ± SE			Spearman Coeff. DI	
	% N. Survey	% Positive Hauls	Tons	Number (Thousand)	10–50	50–100	100–200	200–500	500–800	Shelf	Slope	Overall	Shelf	Slope	Shelf	Slope	Overall	Shelf	Slope
*Tetronarce nobiliana*	40	1.2	18.7 ± 15.7	4 ± 2	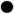	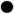	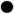	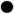	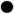	<0.1	1.1 ± 0.9	0.6 ± 0.5	NC	−0.23	0.1 ± 0.1	0.2 ± 0.1	0.1 ± 0.1	NC	−0.26
*Torpedo marmorata*	80	8.2	9.2 ± 2.5	29 ± 5	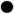	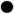	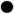	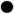	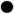	0.6 ± 0.2	0.1 ± 0.0	0.3 ± 0.1	−0.39	0.44	1.6 ± 0.3	0.3 ± 0.1	0.9 ± 0.2	−0.26	0.49
*Torpedo torpedo*	40	0.7	1.1 ± 0.8	3 ± 1	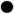			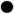	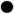	<0.1	<0.1	<0.1	0.27	NC	0.2 ± 0.1	<0.1	0.1 ± 0.0	0.19	NC
*Dipturus batis*	13	0.2	1.4 ± 0.9	1 ± 1				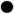	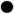	//	0.1 ± 0.1	<0.1	//	NC	//	<0.1	<0.1	//	NC
*Dipturus oxyrinchus*	73	6.3	47.7 ± 11.0	47 ± 19			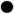	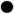	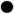	<0.1	2.8 ± 0.7	1.5 ± 0.4	−0.20	0.05	<0.1	2.8 ± 1.1	1.5 ± 0.6	−0.20	0.00
*Leucoraja circularis*	27	0.7	3.6 ± 1.9	2 ± 1					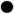	//	0.2 ± 0.1	0.1 ± 0.1	//	0.35	//	0.1 ± 0.1	0.1 ± 0.0	//	0.34
*Leucoraja melitensis*	73	4.9	14.2 ± 4.0	77 ± 25		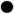	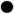	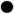	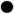	0.2 ± 0.1	0.7 ± 0.2	0.5 ± 0.1	0.24	0.11	0.8 ± 0.3	3.9 ± 1.4	2.5 ± 0.8	0.31	0.07
*Leucoraja naevus*	7	0.1	<0.1	0.1 ± 0.1				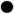		//	<0.1	<0.1	//	NC	//	<0.1	<0.1	//	NC
*Raja asterias*	73	2.5	4.9 ± 1.4	10 ± 2	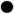	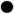		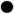		0.3 ± 0.1	<0.1	0.2 ± 0.0	0.31	0.31	0.7 ± 0.2	<0.1	0.3 ± 0.1	0.35	0.31
*Raja brachyura*	7	0.1	0.2 ± 0.2	0.2 ± 0.2					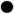	//	<0.1	<0.1	//	0.36	//	<0.1	<0.1	//	0.36
***Raja clavata***	**100**	**18.6**	**304.4 ± 31.8**	**341 ± 51**	** 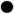 **	** 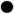 **	** 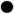 **	** 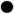 **	** 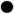 **	**16.5 ± 2.3**	**3.8 ± 0.7**	**9.7 ± 1.0**	0.81 *	**−0.15**	**26.2 ± 5.8**	**4.7 ± 1.1**	**14.6 ± 3.0**	0.86 *	**−0.16**
***Raja miraletus***	**100**	**23.2**	**196.2 ± 18.7**	**1211 ± 125**	** 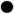 **	** 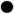 **	** 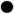 **	** 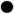 **		**13.3 ± 1.3**	**0.3 ± 0.1**	**6.3 ± 0.6**	0.67 *	**−0.20**	**81.7 ± 8.7**	**1.6 ± 0.8**	**38.6 ± 4.0**	0.58 *	**−0.33**
*Raja montagui*	73	5.2	8.2 ± 1.8	30 ± 6		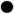	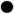	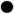		0.5 ± 0.1	0.1 ± 0.0	0.3 ± 0.1	−0.07	−0.12	1.8 ± 0.4	0.2 ± 0.1	1.0 ± 0.2	0.09	0.16
*Raja polystigma*	7	0.1	<0.1	0.1 ± 0.1		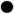				<0.1	//	<0.1	NC	//	<0.1	//	<0.1	NC	//
*Raja radula*	13	0.2	0.1 ± 0.1	1 ± 0.4	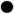	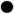				<0.1	//	<0.1	−0.08	//	<0.1	//	<0.1	−0.08	//
*Rostroraja alba*	73	2.5	26.1 ± 9.5	9 ± 2		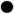	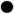	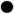	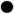	1.6 ± 0.6	0.2 ± 0.2	0.8 ± 0.3	−0.11	−0.41	0.6 ± 0.1	0.1 ± 0.0	0.3 ± 0.1	0.04	−0.39
*Dasyatis pastinaca*	80	2.3	36.9 ± 9.2	9 ± 2	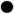	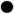	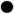			2.5 ± 0.6	//	1.2 ± 0.3	0.36	//	0.7 ± 0.2	//	0.3 ± 0.1	0.36	//
*Pteroplatytrygon violacea*	7	0.1	1.5 ± 1.5	0.3 ± 0.3			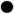			0.1 ± 0.1	//	<0.1	NC	//	<0.1	//	<0.1	NC	//
*Aetomylaeus bovinus*	13	0.1	0.4 ± 0.3	0.5 ± 0.3				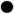		<0.1	<0.1	<0.1	NC	NC	<0.1	<0.1	<0.1	NC	NC
*Myliobatis aquila*	27	0.4	1.5 ± 0.9	1 ± 1	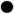	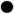				0.1 ± 0.1	//	<0.1	0.21	//	0.1 ± 0.0	//	<0.1	0.31	//

Significant correlations are noted by an asterisk (Spearman coefficients > ±0.50); NC: Not Considered because of the low sample size; //: Not Available. In bold, the species is above the fixed threshold (f% ≥ 10; SSw ≥ 50 tons at least in two surveys). Black dots indicate the depth range within which the species was sampled.

**Table 4:** Synoptic table of batoid abundance sampled during the GRUND survey (autumn) in the GSA15 (Malta Island), from 1990 to 2006. Percent frequency of occurrence for number of surveys (s%) and positive hauls (f%). Standing Stock (SS) expressed in weight (SSw, tons) and number (SSn, thousand). Stratum distribution and mean biomass index kg/km^2^ (BI) expressed by depth of the macrostratum (10–200 m, shelf; 201–800 m, slope; 10–800 m, overall), mean density index N/km^2^ (DI) by depth of the macrostratum, and Spearman coefficients for BI and DI by depth of the macrostratum.

Species	Frequency of Occurrence		SSw (t) and SSn (Mean ± SE)		Stratum Distribution (Depth Interval in m)					BI (kg/km^2^) Mean ± SE			Spearman Coeff. BI		DI (N/km^2^) Mean ± SE			Spearman Coeff. DI	
	% N. Survey	% Positive hauls	Tons	Number (thousand)	10–50	51–100	101–200	201–500	501–800	Shelf	Slope	Overall	Shelf	Slope	Shelf	Slope	Overall	Shelf	Slope
*Tetronarce nobiliana*	20	0.8	1.4 ± 0.8	2 ± 1			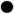	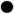	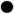	<0.1	0.1 ± 0.0	<0.1	NC	NC	<0.1	0.1 ± 0.1	0.1 ± 0.0	NC	NC
*Torpedo marmorata*	67	10.8	9.0 ± 4.0	24 ± 7	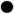	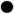	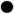	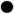		0.8 ± 0.4	<0.1	0.3 ± 0.1	0.1	0.71 *	1.9 ± 0.6	0.1 ± 0.1	0.8 ± 0.2	0.01	0.74 *
*Torpedo torpedo*	7	0.4	1.2 ± 1.2	1 ± 1		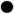	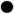			0.1 ± 0.1	//	<0.1	NC	//	0.1 ± 0.1	//	<0.1	NC	//
*Dipturus batis*	7	0.6	13.8 ± 13.8	4 ± 4				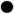		//	0.7 ± 0.7	0.4 ± 0.4	//	NC	//	0.2 ± 0.2	0.1 ± 0.1	//	NC
***Dipturus oxyrichus***	**73**	**19.4**	**283.2 ± 71.5**	**199 ± 43**			** 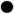 **	** 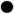 **	** 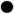 **	**1.6 ± 0.8**	**13.2 ± 3.3**	**9.0 ± 2.3**	0.75 *	0.38 *	**0.5 ± 0.2**	**9.7 ± 2.1**	**6.4 ± 1.4**	0.75 *	**0.22**
*Leucoraja circularis*	40	3.1	9.7 ± 5.5	7 ± 3				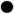	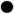	//	0.5 ± 0.3	0.3 ± 0.2	//	0.41	//	0.4 ± 0.1	0.2 ± 0.1	//	0.25
*Leucoraja melitensis*	60	4.7	9.7 ± 4.7	51 ± 22			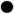	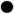	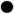	<0.1	0.5 ± 0.2	0.3 ± 0.1	NC	0.29	0.1 ± 0.1	2.5 ± 1.1	1.6 ± 0.7	NC	0.21
*Raja asterias*	13	0.4	0.7 ± 0.7	1 ± 1				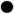	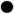	//	<0.1	<0.1	//	NC	//	0.1 ± 0.1	<0.1	//	NC
*Raja brachyura*	7	0.2	0.8 ± 0.8	1 ± 1					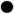	//	<0.1	<0.1	//	NC	//	<0.1	<0.1	//	NC
***Raja clavata***	**100**	**49**	**521.3 ± 67.3**	**627 ± 93**		** 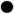 **	** 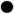 **	** 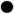 **	** 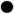 **	**13.0 ± 3.1**	**18.6 ± 3.2**	**16.6 ± 2.1**	0.78 *	**0.12**	**8.1 ± 2.2**	**26.6 ± 4.6**	**20.0 ± 2.9**	0.87 *	**0.09**
***Raja miraletus***	**93**	**27.6**	**135.8 ± 24.1**	**741 ± 156**		** 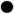 **	** 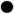 **	** 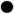 **	** 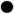 **	**11.6 ± 2.1**	**0.2 ± 0.2**	**4.3 ± 0.8**	**0.16**	**0.23**	**63.4 ± 13.9**	**1.3 ± 1.0**	**23.6 ± 5.0**	**0.18**	**0.34**
*Raja montagui*	47	3.2	4.0 ± 1.6	13 ± 5		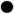		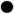		0.2 ± 0.1	0.1 ± 0.1	0.1 ± 0.1	0.50 *	−0.21	0.3 ± 0.3	0.5 ± 0.2	0.4 ± 0.2	0.50 *	−0.21
*Raja radula*	16	2.4	20.5 ± 11.7	55 ± 36	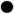	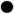	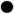			1.8 ± 1.0	//	0.7 ± 0.4	0.75 *	//	4.8 ± 3.2	//	1.7 ± 1.1	0.75 *	//
*Raja undulata*	7	0.1	0.2 ± 0.2	1 ± 1		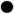				<0.1	//	<0.1	NC	//	0.1 ± 0.1	//	<0.1	NC	//
*Rostroraja alba*	33	1.8	7.1 ± 5.4	6 ± 3				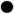	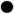	//	0.4 ± 0.3	0.2 ± 0.2	//	0.18	//	0.3 ± 0.1	0.2 ± 0.1	//	0.22
*Dasyatis pastinaca*	47	10.1	108.4 ± 59.5	43 ± 27	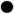	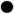	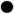			9.6 ± 5.3	//	3.5 ± 1.9	0.56 *	//	3.8 ± 2.4	//	1.4 ± 1.0	0.55 *	//
*Myliobatis aquila*	33	1.4	43.1 ± 23.6	12 ± 6	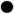	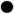				3.8 ± 2.1	//	1.4 ± 0.8	0.64 *	//	1.1 ± 0.6	//	0.4 ± 0.2	0.67 *	//

Significant correlations are noted by an asterisk (Spearman coefficients > ±0.50); NC: Not Considered because of the low sample size; //: Not Available. In bold, the species is above the fixed threshold (f% ≥ 10; SSw ≥ 50 tons at least in two surveys). Black dots indicate the depth range within which the species was sampled.

## Data Availability

The data presented in this study are available on request from the corresponding author.

## Supplementary Materials

The following are available online at https://www.mdpi.com/article/10.3390/ani11082189/s1, Figure S1: Overall positive hauls of *Dipturus oxyrinchus* in the GSA16 and GSA15. Blue and Red crosses indicate, respectively, GRUND (GRU) and MEDITS (MED). The size of the cross is proportional to the number of positive hauls. The dashed areas represent depths below 800 m not explored in the present surveys., Figure S2: Overall positive hauls of *Raja clavata* in the GSA16 and GSA15. Blue and Red crosses indicate, respectively, GRUND (GRU) and MEDITS (MED). The size of the cross is proportional to the number of positive hauls. The dashed areas represent depths below 800 m not explored in the present surveys, Figure S3: Overall positive hauls of *Raja miraletus* in the GSA16 and GSA15. Blue and Red crosses indicate, respectively, GRUND (GRU) and MEDITS (MED). The size of the cross is proportional to the number of positive hauls. The dashed areas represent depths below 800 m not explored in the present surveys. Table S1:Calendar of the MEDITS (MED) survey carried out in the GSA16 and GSA15. Blue color depicted the surveys timeframe, Table S2. Calendar of the GRUND (GRU) survey carried out in the GSA16 and GSA15. Blue color depicted the surveys timeframe, **Table S3**: Synopsis of the batoids life history traits estimated from the South of Sicily compared to the other Mediterranean areas. Table S4. Taxonomic and systematic features of the 38 species of batoids documented in the Mediterranean Sea following Dulvy et al. [11], integrated by Last et al. [137], and Fricke et al. [138]. In the Family column, the most accepted common/vernacular name is also presented, Video S1: not applicable. References [21,22,30,31,33,34,66,67,71,72,73,74,75,76,77,78,79,80,81,83,84,85,87,88,91,92,93,94,96,97,98,99,100,101,102,104,105,107,108,109,110,111,112,113,114,115,116,117,118,120,121,122,123,124,125,126,127,128,139,140,141,142,143,144] are cited in the supplementary materials.
