# Supplementary material for: Batoid Abundances, Spatial Distribution, and Life History Traits in the Strait of Sicily (Central Mediterranean Sea): Bridging a Knowledge Gap through Three Decades of Survey
Source: Animals (Basel). 2021 Jul 23;11(8):2189. doi: 10.3390/ani11082189 (PMC8388512; doi:10.3390/ani11082189)
Supplement: Supplementary file 1 [file animals-11-02189-s001.zip › Table_S2.pdf]

GRUND GSA16									
YEAR	N° HAULS	September		October		November		December	
		1–15	16–30	1–15	16–31	1–15	16–30	1–15	16–31
1990	53								
1991	70								
1992	72								
1993	No cruise								
1994	83								
1995	90								
1996	60								
1997	67								
1998	62								
1999	No cruise								
2000	53								
2001	53								
2002	48								
2003	48								
2004	47								
2005	126								
2006	132								

GRUND GSA15									
YEAR	N° HAULS	September		October		November		December	
		1–15	16–30	1–15	16–31	1–15	16–30	1–15	16–31
1990	12								
1991	7								
1992	20								
1993	No cruise								
1994	25								
1995	16								
1996	15								
1997	29								
1998	29								
1999	No cruise								
2000	29								
2001	34								
2002	13								
2003	47								
2004	45								
2005	30								
2006	30								
