# Supplementary material for: Batoid Abundances, Spatial Distribution, and Life History Traits in the Strait of Sicily (Central Mediterranean Sea): Bridging a Knowledge Gap through Three Decades of Survey
Source: Animals (Basel). 2021 Jul 23;11(8):2189. doi: 10.3390/ani11082189 (PMC8388512; doi:10.3390/ani11082189)
Supplement: Supplementary file 1 [file animals-11-02189-s001.zip › Table_S3.pdf]

Species	TL max (mm)	b	k	sex	sex ratio (F/M)	L_{50} (mm)	L_∞ (mm)	K ($year^{-1}$)	t_0 (year)	Area	References
Tetronarce nobiliana	970	//	//	F	0.64:1	//	//	//	//	South of Sicily, Italy	Present study
	770	//	//	M		//	//	//	//	South of Sicily, Italy	Present study
	//	2.89	3.3^{10-5}	C		//	//	//	//	South of Sicily, Italy	Present study
	1800	//	//	C	//		//	//	//	Mediterranean	Serena [123]
	1200	//	//	F	0.95:1	600	//	//	//	Tunisia	Capapè et al. [139]
	770	//	//	M		550	//	//	//	Tunisia	Capapè et al. [139]
	1015	//	//	F	//	//	//	//	//	Sardinia, Italy	Bellodi et al. [74]
	592	//	//	M	//	//	//	//	//	Sardinia, Italy	Bellodi et al. [74]
	//	3.1	0.015	C	0.44:1	//	//	//	//	Turkey	Başusta et al. [66]
	//	3.27	7.8^{10-3}	F	1.38:1	//	//	//	//	Turkey	Kaya & Başusta [67]
	//	2.74	0.042	M		//	//	//	//	Turkey	Kaya & Başusta [67]
	355	3.1	0.015	C		//	744.7	0.109	−1.058	Turkey	Kaya & Başusta [67]
Torpedo marmorata	495	2.96	2.7^{10-7}	F	0.85:1	//	//	//	//	South of Sicily, Italy	Present study
	370	2.67	1.0^{10-4}	M		//	//	//	//	South of Sicily, Italy	Present study
	//	2.85	4.5^{10-5}	C		//	//	//	//	South of Sicily, Italy	Present study
	//	//	//	F	//	400	//	//	//	Mediterranean	Serena [123]
	//	//	//	M	//	290	//	//	//	Mediterranean	Serena [123]
	560	2.92	3^{10-5}	F	1.18:1	379.4	622	0.15	//	Sardinia, Italy	Bellodi et al. [74]
	432	2.93	4^{10-5}	M		272.2	485	0.14	−2.28	Sardinia, Italy	Bellodi et al. [74]
	400	3.04	0.017	F	1.09:1	//	//	//	//	Turkey	Duman & Başusta [71]
	303	2.82	0.031	M		//	//	//	//	Turkey	Duman & Başusta [71]
	//	2.99	0.019	C		//	573.2	0.187	−0.392	Turkey	Duman & Başusta [71]
	612	//	//	F	1.41:1	355	//	//	//	Egypt	Abdel-Aziz [77]
	395	//	//	M		255	//	//	//	Egypt	Abdel-Aziz [77]
	553	2.85	4^{10-4}	F	0.71:1	312	//	//	//	Latium, Italy	Consalvo et al. [75]
	364	2.72	9^{10-4}	M		251	//	//	//	Latium, Italy	Consalvo et al. [75]
	865	//	//	F	0.81:1	//	//	//	//	Turkey	Yeldan & Gundogdu [72]
	722	//	//	M		//	//	//	//	Turkey	Yeldan & Gundogdu [72]
	//	2.74	0.012	C		//	//	//	//	Turkey	Yeldan & Gundogdu [72]
	340	2.91	2.7^{10-7}	C	//	//	//	//	//	Turkey	Filiz & Bilge [73]
	310	1.75	1.337	F	0.37:1	//	//	//	//	Lebanon	Lteif et al. [76]
	300	2.12	0.352	M		//	//	//	//	Lebanon	Lteif et al. [76]
Torpedo torpedo	275	//	//	F	1.86:1	//	//	//	//	South of Sicily, Italy	Present study
	355	//	//	M		//	//	//	//	South of Sicily, Italy	Present study
	//	3.13	0.015	C		//	//	//	//	South of Sicily, Italy	Present study
	435	//	//	F	0.61:1	//	//	//	//	Tunisia	El Kamel-Moutalibi et al. [78]

	403	//	//	M		//	//	//	//	Tunisia	El Kamel-Moutalibi et al. [78]
	477	3.86	3^{10-4}	F	1.32:1	//	//	//	//	Latium, Italy	Consalvo et al. [75]
	445	2.74	6^{10-4}	M		//	//	//	//	Latium, Italy	Consalvo et al. [75]
	437	//	//	F	1:1	//	//	//	//	Sardinia, Italy	Bellodi et al. [74]
	437	//	//	M		//	//	//	//	Sardinia, Italy	Bellodi et al. [74]
	408	//	//	F	0.76:1	//	//	//	//	Egypt	Abdel-Aziz [77]
	391	//	//	M		//	//	//	//	Egypt	Abdel-Aziz [77]
Dipturus batis	1260	//	//	F	//	//	//	//	//	South of Sicily, Italy	Present study
	2500	//	//	C	//	//	//	//	//	Mediterranean	Serena et al. [79]
	2850	//	//	C	//	//	//	//	//	Mediterranean	Serena et al. [80]
Dipturus nidarosiensis	956	//	//	M	//	//	//	//	//	South of Sicily, Italy	Present study
	1482	//	//	F	//	//	//	//	//	Sardinia, Italy	Cannas et al. [81]
	1180	//	//	M	//	//	//	//	//	Sardinia, Italy	Cannas et al. [81]
	1422	//	//	F	//	//	//	//	//	Apulia, Italy	Carbonara et al. [83]
	1235	//	//	M	//	//	//	//	//	Apulia, Italy	Carbonara et al. [83]
	680	//	//	F	//	//	//	//	//	Adriatic Sea, Italy	Isajlović et al. [84]
	725	//	//	M	//	//	//	//	//	Spain	Ramírez-Amaro et al. [85]
Dipturus oxyrinchus	1100	3.29	5.4^{-07}	F	1.56:1	695	1365	0.11	//	South of Sicily, Italy	Present study
	1240	3.20	1.0^{-06}	M		860	1240	0.26	//	South of Sicily, Italy	Present study
	//	3.25	7.2^{10-7}	C		//	//	//	//	South of Sicily, Italy	Present study
	1500	//	//	C	//	//	//	//	//	Mediterranean	Serena et al. [79]
	1175	//	//	F	//	1035	1369.4	0.14	//	Sardinia, Italy	Bellodi et al. [87]
	995	//	//	M	//	910	1141.4	0.16	//	Sardinia, Italy	Bellodi et al. [87]
	//	//	//	C	//		1275.5	0.14	//	Sardinia, Italy	Bellodi et al. [87]
	//	3.34	9^{10-4}	F	0.99:1	830	//	//	//	Turkey	Yıgın and Ismen [88]
	//	3.37	8^{10-4}	M		650	//	//	//	Turkey	Yıgın and Ismen [88]
	//	3.35	8^{10-4}	C		//	2565	0.04	−1.17	Turkey	Yıgın and Ismen [88]
		3.40	0.001	C	//	//	//	//	//	Turkey	Filiz & Bilge [73]
		3.28	1.2^{10-2}	F	1.49:1	795	//	//	//	Syria	Alkusairy & Saad [80]
		3.28	5^{10-3}	M		709	//	//	//	Syria	Alkusairy & Saad [80]
		3.35	9^{10-3}	C		//	//	//	//	Syria	Alkusairy & Saad [80]
	1050	//	//	F	0.72:1	821	1239	0.08	−1.26	Tunisia	Kadri et al. [31]
	950	//	//	M		720.5	1201	0.12	−1.18	Tunisia	Kadri et al. [31]
Leucoraja circularis	880	//	//	F	//	//	//	//	//	South of Sicily, Italy	Present study
	750	//	//	M	//	//	//	//	//	South of Sicily, Italy	Present study
	1200	//	//	C	//	//	//	//	//	Mediterranean	Serena et al. [79]; Ebert & Stehmann [91]
	820	3.08	0.04	C	//	//	//	//	//	Turkey	Deval et al. [92]
	880	//	//	F	1.2:1	//	//	//	//	Tunisia	Mnasri et al. [93]
	672	//	//	M		//	//	//	//	Tunisia	Mnasri et al. [93]
Leucoraja fullonica	1200	//	//	C	//	//	//	//	//	Mediterranean	Serena et al. [79]; Ebert & Stehmann [91]

Leucoraja melitensis	450	//	//	F	//	//	//	//	//	South of Sicily, Italy	Present study
	520	//	//	M	//	//	//	//	//	South of Sicily, Italy	Present study
	//	3.12	2.4^{10-6}	C	//	//	//	//	//	South of Sicily, Italy	Present study
	420	//	//	F	//	//	//	//	//	Strait of Sicily, Italy	Ragonese et al. [22]
	500	//	//	//	//	//	//	//	//	Mediterranean	Serena et al. [79]; Stehmann & Bürkel [94]
Leucoraja naevus	750	//	//	F	//	//	//	//	//	Mediterranean	Ebert & Stehmann [91]
	680	//	//	M	//	//	//	//	//	Mediterranean	Ebert & Stehmann [91]
	700	//	//	C	//	//	//	//	//	Mediterranean	Serena et al. [79]
Raja asterias	590	3.14	2.4^{10-6}	F		//	//	//	//	South of Sicily, Italy	Present study
	550	3.02	4.9^{10-6}	M	1.13:1	//	//	//	//	South of Sicily, Italy	Present study
	//	3.09	3.2^{10-6}	C		//	//	//	//	South of Sicily, Italy	Present study
	680	//	//	//	//	//	674.5	0.45	−0.23	Tuscany, Italy	Bono et al. [96]
	800	//	//	//	//	//	//	//	//	Mediterranean	Serena et al. [79]
	//	3.32	0.002	F	//	600	760	0.41	0	Mediterranean	Serena & Abella [97]
	//	3.01	0.006	M	//	561	725	0.42	0	Mediterranean	Serena & Abella [97]
	//	3.39	0.001	F	//	//	//	//	//	Sardinia, Italy	Cau et al. [98]
	//	3.33	0.001	M	//	//	//	//	//	Sardinia, Italy	Cau et al. [98]
	//	3.39	0.002	C	//	//	//	//	//	Sardinia, Italy	Cau et al. [98]
	//	3.13	0.003	C	//	//	//	//	//	W Ionian Sea, Italy	Carbonara et al. [99]
	//	3.48	0.001	C	//	//	//	//	//	N Tyrrhenian Sea, Italy	De Ranieri [100]
	//	//	//	F	1.07:1	561	//	//	//	Tuscany, Italy	Barone et al. [140]
	//	//	//	M		517	//	//	//	Tuscany, Italy	Barone et al. [140]
	//	//	//	//	1:1	//	//	//	//	Central Tyrrhenian Sea	Minervini et al. [101]
	//	//	//	//	1:1	//	//	//	//	Tuscany, Italy	Abella et al. [102]
Raja brachyura	410	//	//	F	0.85:1	//	//	//	//	South of Sicily, Italy	Present study
	320	//	//	M		//	//	//	//	South of Sicily, Italy	Present study
	1200	//	//	C	//	//	//	//	//	Mediterranean	Serena et al. [79]
	735	3.40	5^{10-7}	F	0.83:1					Sardinia, Italy	Catalano et al. [104]
	955	3.28	1^{10-6}	M						Sardinia, Italy	Catalano et al. [104]
	1055	//	//	F	1.08:1	872	1111.4	0.10	-1.3	Sardinia, Italy	Porcu et al. [105]
	965	//	//	M		808	1088.1	0.11	-1.2	Sardinia, Italy	Porcu et al. [105]
Raja clavata	920	//	//	F		635	1260	0.16	//	South of Sicily, Italy	Present study
	1100	//	//	M	1:1	574	1100	0.26	//	South of Sicily, Italy	Present study
	//	//	//	C		//	//	//	//	South of Sicily, Italy	Present study

	920	//	//	F	1.08:1	//	1260	0.098	-0.512	Strait of Sicily, Italy	Cannizzaro et al. [21]
	780	//	//	M		//	1167	0.106	-0.412	Strait of Sicily, Italy	Cannizzaro et al. [21]
	1100	3.40	0.001	F	1.41:1	814	1146	0.11	1.23	Tunisia	Kadri et al. [32]
	890	3.23	0.002	M		674	1008	0.14	1.13	Tunisia	Kadri et al. [32]
		2.64*	0.023		1.04:1	//	//	//	//	Turkey	Başusta et al. [66]
	1300	//	//	C	//	//	//	//	//	Mediterranean	Serena et al. [79]
	//	3.21	0.003	F	1:1	//	//	//	//	Balearic Island, Spain	Ramírez-Amaro et al. [107]
	//	3.05	0.005	M		//	//	//	//	Balearic Island, Spain	Ramírez-Amaro et al. [107]
	//	//	//	F		783	//	//	//	Tuscany, Italy	Barone [108]
	//	//	//	M		663	//	//	//	Tuscany, Italy	Barone [108]
	//	//	//	F	1.32:1	930	//	//	//	Turkey	Saglam & Ak [109]
	//	//	//	M		920	//	//	//	Turkey	Saglam & Ak [109]
	//	//	//	F	1.52:1	754.4	//	//	//	Black Sea	Bilgin & Onay [110]
	//	//	//	M		717.7	//	//	//	Black Sea	Bilgin & Onay [110]
	//	//	//	F		612	//	//	//	Adriatic Sea, Italy	Krstulović Šifner et al. [111]
	//	//	//	M		593	//	//	//	Adriatic Sea, Italy	Krstulović Šifner et al. [111]
	//	//	//	F	1.38:1		988.9	//	//	Adriatic Sea, Italy	Carbonara et al. [112]
	//	//	//	M			941.0	//	//	Adriatic Sea, Italy	Carbonara et al. [112]
	990	3.30	0.002	C	//	//	//	//	//	Turkey	Filiz & Bilge [73]
	//	3.20	0.003	C	//	//	//	//	//	Lebanon	Lteif et al. [76]
	//	2.78	0.018	F	//	//	//	//	//	Albania	Bakiu et al. [141]
	//	2.64	0.032	M	//	//	//	//	//	Albania	Bakiu et al. [141]
	903	2.72	0.023	C	//	//	//	//	//	Albania	Bakiu et al. [141]
	470	3.50	2.7^{10-7}	F		364	840	0.36	//	South of Sicily, Italy	Present study
	460	3.42	4.2^{10-7}	M	0.96:1	349	800	0.41	//	South of Sicily, Italy	Present study
	//	3.46	3.4^{10-7}	C		//	//	//	//	South of Sicily, Italy	Present study
	540	3.26*	0.002	C	1:1	//	//	//	//	Turkey	Başusta et al. [66]
	560	//	//	F	1.12:1	343	692	0.18	-0.11	Tunisia	Kadri et al. [30]
	580	//	//	M		418	670	0.22	-1.01	Tunisia	Kadri et al. [30]
	560	3.39	0.001	F	1.25:1	//	//	//	//	Tunisia	Kadri et al. [33]
	580	3.31	0.001	M		//	//	//	//	Tunisia	Kadri et al. [33]
Raja miraletus	600	//	//	C	//	//	//	//	//	Mediterranean	Serena [123]
	510	3.43	0.001	F		423	//	//	//	Adriatic Sea, Italy	Ungaro [113]
	495		//	M	1.39:1	364	//	//	//	Adriatic Sea, Italy	Ungaro [113]
	//		//	C		//	//	//	//	Adriatic Sea, Italy	Ungaro [113]
	//	4.15	1^{10-4}	C	//	//	//	//	//	Turkey	Filiz & Bilge [73]
	//	2.12	1.6^{10-3}	C	//	//	//	//	//	Lebanon	Lteif et al. [76]
	//	//	//	F	1.39:1		919	0.172	-0.24	Egypt	Abdel-Aziz [114]
	//	//	//	M	//	//	879	0.193	-0.50	Egypt	Abdel-Aziz [114]
	//	//	//	C	//	//	910	0.180	-0.36	Egypt	Abdel-Aziz [114]
	//	//	//	F	//	440	//	//	//	Mediterranean	Serena & Abella [97]
	//	//	//	M	//	400	//	//	//	Mediterranean	Serena & Abella [97]

Raja montagui	520	3.51	2.8^{10-7}	F		//	//	//	//	South of Sicily, Italy	Present study
	495	3.55	2.0^{10-7}	M	1.22:1	//	//	//	//	South of Sicily, Italy	Present study
	//	3.50	2.8^{10-7}	C		//	//	//	//	South of Sicily, Italy	Present study
	800	//	//	C	//	//	//	//	//	Mediterranean	Serena et al. [80]
Raja polystigma	405	//	//	F		//	//	//	//	South of Sicily, Italy	Present study
	475	//	//	M	0.64:1	//	//	//	//	South of Sicily, Italy	Present study
	//	3.45	4.4^{10-7}	C		//	//	//	//	South of Sicily, Italy	Present study
	600	//	//	C	//	//	//	//	//	Mediterranean	Serena et al. [79]
	590	//	//	F	1.12:1	//	691	0.26	-1.718	Sardinia, Italy	Bellodi et al. [115]
	521	//	//	M		//	748	0.11	//	Sardinia, Italy	Bellodi et al. [115]
	590	//	//	F		506	681	0.28	//	Sardinia, Italy	Porcu et al. [116]
	515	//	//	M	1.03:1	488	596	0.32	//	Sardinia, Italy	Porcu et al. [116]
	//	//	//	C		//	691	0.26	//	Sardinia, Italy	Porcu et al. [116]
	//	//	//	F	//	63	//	//	//	Tunisia	Capapè & Quignard [142]
	//	//	//	M	//	53	//	//	//	Tunisia	Capapè & Quignard [142]
Raja radula	440	//	//	F	0.51:1	//	//	//	//	South of Sicily, Italy	Present study
	340	//	//	M		//	//	//	//	South of Sicily, Italy	Present study
	800	3.35	0.011	F		565	972	0.15	0.35	Tunisia	Kadri et al. [34]
	700	3.32	0.017	M	1.38:1	47	764	0.22	0.16	Tunisia	Kadri et al. [34]
	//	3.34	0.014	C			864	0.19	0.25	Tunisia	Kadri et al. [34]
	610	3.36	0.002	F		460	829	0.16	-0.59	Aegean Sea	Yığın & Ismen [117]
	540	3.30	0.002	M	1.29:1	400	747	0.20	-0.22	Aegean Sea	Yığın & Ismen [117]
	//	3.35	0.002	C			1077	0.09	-1.40	Aegean Sea	Yığın & Ismen [117]
	590	3.41	0.001	C	//	//	//	//	//	Turkey	Deval et al. [143]
	//	2.99*	0.021	F		//	//	//	//	South Eeast Sicily, Italy	Tiralongo et al. [118]
	//	2.92*	0.027	M	1.05:1	//	//	//	//	South Eeast Sicily, Italy	Tiralongo et al. [118]
	//	2.94*	0.025	C		//	//	//	//	South Eeast Sicily, Italy	Tiralongo et al. [118]
Raja undulata	1200	//	//	C	//	//	//	//	//	Mediterranean	Serena et al. [79]
Rostroraja alba	1450	3.18	1.9^{10-6}	F		//	//	//	//	South of Sicily, Italy	Present study
	1320	3.09	1.7^{10-6}	M	0.89	//	//	//	//	South of Sicily, Italy	Present study
	//	3.14	1.7^{10-6}	C		//	//	//	//	South of Sicily, Italy	Present study
	1600	3.61	0.008	F	0.78:1	//	//	//	//	Tunisia	Kadri et al. [35]
	1500	3.53	0.001	M		//	//	//	//	Tunisia	Kadri et al. [35]
	1590	//	//	F		800	3390	0.04	-0.28	Turkey	Yıgın & Ismen [120]
	1350	//	//	M	1.13:1	750	3060	0.04	-0.31	Turkey	Yıgın & Ismen [120]
	//	3.27	0.002	C		//	3110	0.04	-0.25	Turkey	Yıgın & Ismen [120]

Dasyatis pastinaca	970	//	//	F		//	//	//	//	South of Sicily, Italy	Present study
	710	//	//	M	1.94:1	//	//	//	//	South of Sicily, Italy	Present study
	//	2.48	3^{10-3}	C		//	//	//	//	South of Sicily, Italy	Present study
	2500	//	//	C	//	//	//	//	//	Mediterranean	Serena [123]
	//	3.32	0.042	C	1.14:1	//	//	//	//	Turkey	Başusta et al. [66]
	934	2.93	0.039	F		//	//	//	//	Turkey	Yeldan & Gundogdu [72]
	689	3.32	0.002	M	1.32:1	//	//	//	//	Turkey	Yeldan & Gundogdu [72]
				C			583*	0.06	-0.213	Turkey	Yeldan & Gundogdu [72]
	//	2.81	0.015	C	//	//	//	//	//	Turkey	Filiz & Bilge [73]
	1300	3.23	0.002	C	//	//	//	//	//	Turkey	Özbek et al. [121]
	//	3.06*	0.027	F		//	1271*	0.058	-1.51	Turkey	Girgin & Basusta [122]
	//	3.08*	0.025	M	1.19:1	//	1145*	0.041	-3.63	Turkey	Girgin & Basusta [122]
	693	3.08*	0.025	C		//	1044*	0.075	-1.43	Turkey	Girgin & Basusta [122]
	1009			F		//	2198	0.041	-2.61	Turkey	Yeldan et al. [124]
	695			M	1.29:1	//	2031	0.039	-2.00	Turkey	Yeldan et al. [124]
						//	2944	0.029	-2.2	Turkey	Yeldan et al. [124]
	1140	3.51	8^{10-4}	F		//	1200	0.086	-1.24	Turkey	Yıgın & Ismen [125]
	1100	3.61	5^{10-4}	M	1.62:1	//	1885	0.065	-0.04	Turkey	Yıgın & Ismen [125]
				C		625	1860	0.051	-1.40	Turkey	Yıgın & Ismen [125]
	880	3.17	2.4^{10-3}	F		460	//	//	//	Turkey	Ismen [126]
	730	3.44	9^{10-4}	M	0.75:1	430	//	//	//	Turkey	Ismen [126]
	//	3.31	0.001	C		//	1215	0.089	-1.615	Turkey	Ismen [126]
	690*	//	//	F	1.24:1	40*	//	//	//	Tunisia	Saadaoui et al. [127]
	470*	//	//	M		33*	//	//	//	Tunisia	Saadaoui et al. [127]
Pteroplatytrygon violacea	1060	//	//	F	//	//	//	//	//	South of Sicily, Italy	Present study
	610	//	//	F	1.43:1	//	//	//	//	Mediterranean	Hemida et al. [144]
	580	//	//	M		//	//	//	//	Mediterranean	Hemida et al. [144]
Aetomylaeus bovinus	2660	//	//	F	0.96:1	//	//	//	//	South of Sicily, Italy	Present study
	830	//	//	M		//	//	//	//	South of Sicily, Italy	Present study
	//	2.90*	0.019	C	0.83:1	//	//	//	//	Turkey	Başusta et al. [66]
	//	2.90*	0.065	F		//	2426	0.056	-1.904	Turkey	Başusta & Aslan [128]
	//	3.09*	0.035	M	1:1	//	2384	0.044	-2.982	Turkey	Başusta & Aslan [128]
	1292	2.94*	0.058	C		//	1621	//	3.094	Turkey	Başusta & Aslan [128]
Myliobatis aquila	1110	//	//	F	//	//	//	//	//	South of Sicily, Italy	Present study
	775	//	//	M	//	//	//	//	//	South of Sicily, Italy	Present study
	//	4.01	4^{10-9}	C	//	//	//	//	//	South of Sicily, Italy	Present study
	2600	//	//	C	//	//	//	//	//	Mediterranean	Serena [123]
	//	3.34	0.001	C	//	//	//	//	//	Turkey	Filiz & Bilge [73]

*expressed as Disc Width (DW). //: Not Available

References

21. Cannizzaro, L.; Garofalo, G.; Levi, D.; Rizzo, P.; Gancitano, S. *Raja clavata* (Linneo, 1758) nel Canale di Sicilia: Crescita, distribuzione e abbondanza. *Biol. Mar. Mediterr.* **1995**, *2*, 257–262. (In Italian)
22. Ragonese, S.; Cigala Fulgosi, F.; Bianchini, M.L.; Norrito, G.; Sinacori, G. Annotated check list of the skates (Chondrichthyes, Rajidae) in the Strait of Sicily (Central Mediterranean). *Biol. Mar. Mediterr.* **2003**, *10*, 874–881. (In Italian)
30. Kadri, H.; Marouani, S.; Sadi, B.; Bradai, M.N.; Ghorbel, M.; Bouan, A.; Morize, E. Age, growth and reproduction of *Raja miraletus* (Linnaeus, 1758) (Chondrichthyes: Rajidae) of the Gulf of Gabès (Tunisia, Central Mediterranean Sea). *Mar. Biol. Res.* **2012**, *8*, 388–396, doi:10.1080/17451000.2011.619546.
31. Kadri, H.; Marouani, S.; Bradai, M.N.; Bouaïn, A.; Morize, E. Age, growth, longevity, mortality and reproductive biology of *Dipturus oxyrinchus*, (Chondrichthyes: Rajidae) off the Gulf of Gabès (Southern Tunisia, central Mediterranean). *J. Mar. Biol. Assoc. UK* **2014**, *95*, 569–577, doi:10.1017/s0025315414000551.
33. Kadri, H.; Marouani, S.; Bradai, M.N.; Bouaïn, A.; Morize, E. Distribution and Morphometric Characters of the Mediterranean Brown Ray, *Raja miraletus* (Chondrichthyes: Rajidae) in the Gulf of Gabes (Tunisia, Central Mediterranean). *Am. J. Agric. For.* **2014**, *2*, 45–50, doi:10.11648/j.ajaf.20140202.15.
34. Kadri, H.; Marouani, S.; Bradai, M.N.; Bouaïn, A.; Morize, E. Age, growth and length-weight relationship of the rough skate, *Raja radula* (Linnaeus, 1758) (Chondrichthyans: Rajidae), from the Gulf of Gabes (Tunisia, Central Mediterranean). *J. Coast. Life Med.* **2014**, *2*, 344–349.
66. Başusta, A.; Başusta, N.; Sulikowski, J.A.; Driggers, W.B.; Demirhan, S.A.; Çiçek, E. Length-weight relationships for nine species of batoids from the Iskenderun Bay, Turkey. *J. Appl. Ichthyol.* **2012**, *28*, 850–851, doi:10.1111/j.1439-0426.2012.02013.x.
67. Kaya, G.; Başusta, N. A study on age and growth of juvenile and semi adult *Torpedo nobiliana* Bonaparte, 1835 inhabiting Iskenderun Bay, northeastern Mediterranean Sea. *Acta Biol. Turc.* **2016**, *29*, 143–149.
71. Duman, Ö.V.; Başusta, N. Age and growth characteristics of marbled electric ray *Torpedo marmorata* (Risso, 1810) inhabiting Iskenderun Bay, North-eastern Mediterranean Sea. *Turk. J. Fish Aquat. Sci.* **2013**, *13*, 541–549, doi:10.4194/1303-2712-v13_3_19.
72. Yeldan, H.; Gundogdu, S. Morphometric relationships and growth of common stingray, *Dasyatis pastinaca* (Linnaeus, 1758) and marbled stingray, *Dasyatis marmorata* (Steindachner, 1892) in the northeastern Levantine Basin. *J. Black Sea/Mediterr. Environ.* **2018**, *24*, 10–27.
73. Filiz, H.; Bilge, G. Length-weight relationships of 24 fish species from the North Aegean Sea, Turkey. *J. Appl. Ichthyol.* **2004**, *20*, 431–432, doi:10.1111/j.1439-0426.2004.00582.x.
74. Bellodi, A.; Mulas, A.; Carbonara, P.; Cau, A.; Cuccu, D.; Marongiu, M.F.; Mura, V.; Pesci, P.; Zupa, W.; Porcu, C.; et al. New insights into life–history traits of Mediterranean Electric rays (Torpediniformes: Torpedinidae) as a contribution to their conservation. *Zoology* **2021**, *146*, 125922, doi:10.1016/j.zool.2021.125922.
75. Consalvo, I.; Scacco, U.; Romanelli, M.; Vacchi, M. Comparative study on the reproductive biology of *Torpedo torpedo* (Linnaeus, 1758) and *T. marmorata* (Risso, 1810) in the central Mediterranean *Sea. Sci. Mar.* **2007**, *71*, 213–222, doi:10.3989/scimar.2007.71n2213.
76. Lteif, M.; Mouawad, R.; Jemaa, S.; Khalaf, G.; Lenfant, P.; Verdoit-Jarraya, M. The length-weight relationships of three sharks and five batoids in the Lebanese marine waters, eastern Mediterranean. *Egypt. J. Aquat. Res.* **2016**, *42*, 475–477, doi:10.1016/j.ejar.2016.09.008.
77. Abdel-Aziz, S. Observations on the biology of the common torpedo (*Torpedo torpedo*, Linnaeus, 1758) and marbled electric ray (*Torpedo marmorata*, Risso, 1810) from Egyptian Mediterranean waters. *Mar. Freshw. Res.* **1994**, *45*, 693–704, doi:10.1071/mf9940693.
78. El Kamel-Moutalibi, O.; Mnasri, N.; Boumaïza, M.; Ben Amor, M.M.; Reynaud, C.; Capapé, C. Maturity, reproductive cycle and fecundity of common torpedo, *Torpedo torpedo* (Chondrichthyes, Torpedinidae) from the Lagoon of Bizerte (Northeastern Tunisia, central Mediterranean). *J. Ichthyol.* **2013**, *53*, 758–774, doi:10.1134/s0032945213060118.
79. Serena, F.; Mancusi, C.; Barone, M. (Eds.) Field guide to the identification of the skates (Rajidae) of the Mediterranean Sea. Guidelines for data collection and analysis. *Biol. Mar. Mediterr.* **2010**, *17*, 1–204.
80. Serena, F.; Mancusi, C.; Barone, M. MEDiterranean Large Elasmobranchs Monitoring. In *Protocollo di Acquisizione Dati*; SharkLife program: Roma, Italy, 2014; 130p. (In Italian)
81. Cannas, R.; Follesa, M.C.; Cabiddu, S.; Porcu, C.; Salvadori, S.; Iglésias, S.P.; Deiana, A.M.; Cau, A. Molecular and morphological evidence of the occurrence of the Norwegian skate *Dipturus nidarosiensis* (Storm, 1881) in the Mediterranean Sea. *Mar. Biol. Res.* **2010**, *6*, 341–350, doi:10.1080/17451000903428496.
83. Fang, K.; Chen, L.; Zhou, J.; Yang, Z.P.; Dong, X.F.; Zhang, H.B. On the presence of *Dipturus nidarosiensis* (Storm, 1881) in the Central Mediterranean area. *PeerJ* **2019**, *7*, e7009, doi:10.7717/peerj.7009.
84. Isajlović, I.; Dragičević, B.; Manfredi, C.; Vrgoč, N.; Piccinetti, C.; Dulčić, J. Additional records of Norwegian skate *Dipturus nidarosiensis* (Storm, 1881) (Pisces: Rajidae) in the Adriatic Sea. *Acta Adriat.* **2020**, *61*, 217–222, doi:10.32582/aa.61.2.11.
85. Ramírez-Amaro, S.; Ordines, F.; Puerto, M.Á.; García, C.; Ramon, C.; Terrasa, B.; Massutí, E. New morphological and molecular evidence confirm the presence of the Norwegian skate *Dipturus nidarosiensis* (Storm, 1881) in the

Mediterranean Sea and extend its distribution to the western basin. *Mediterr. Mar. Sci.* **2017**, *18*, 251–259, doi:10.12681/mms.1950.
87. Bellodi, A.; Porcu, C.; Cannas, R.; Cau, A.; Marongiu, M.F.; Mulas, A.; Vittori, S.; Follesa, M.C. Life-history traits of the long-nosed skate *Dipturus oxyrinchus*. *J. Fish Biol.* **2016**, *90*, 867–888, doi:10.1111/jfb.1320.
88. Yığın, C.Ç.; Ismen, A. Age, growth, reproduction and feed of longnosed skate, *Dipturus oxyrinchus* (Linnaeus, 1758) in Saros Bay, the north Aegean Sea. *J. Appl. Ichthyol.* **2010**, *26*, 913–919, doi:10.1111/j.1439-0426.2010.01510.x.
91. Ebert, D.A.; Stehmann, M.F. *Sharks, Batoids and Chimaeras of the North Atlantic*; FAO: Roma, Italia, 2013.
92. Deval, M.C.; Güven, O.; Saygu, İ.; Kabapçioğlu, T. Length-weight relationships of 10 fish species found off Antalya Bay, eastern Mediterranean. *J. Appl. Ichthyol.* **2013**, *30*, 567–568, doi:10.1111/jai.12382.
93. Mnasri, N.; Boumaïza, M. Capapé. Morphological data, biological observations and occurrence of a rare skate, *Leucoraja circularis* (Chondrichthyes: Rajidae), off the northern coast of Tunisia (central Mediterranean). *Pan-Am. J. Aquat. Sci.* **2009**, *4*, 70–78.
94. Stehmann, M.; Bürkel, D.L. Rajidae. *Fishes of the Northeastern Atlantic and Mediterranean*; Whitehead, P.J.P., Bauchot, M.L., Hureau, J.C., Nielsen, J., Tortonese, E. Eds.; UNESCO: Paris, France, 1984. p. 163–196.
96. Bono, L.; De Ranieri, S.; Fabiani, O.; Mancusi, C.; Serena, F. Studio sull'accrescimento di *Raja asterias* (Delaroche, 1809) (Chondrichthyes, Raidae) attraverso l'analisi delle vertebre. *Biol. Mar. Mediterr.* **2005**, *12*, 470–474. (In Italian)
97. Serena, F.; Abella, A. Sintesi delle conoscenze sulle risorse da pesca dei fondi del Mediterraneo centrale (Italia e Corsica). *Biol Mar. Medit.* **1999**, *6*, 82–86.
98. Cau, A.; Follesa, M.C.; Pesci, P.; Cuccu, D.; Sabatini, A. Campionamento Biologico Delle Catture. Sezioni C ed E. Dipartimento di Scienze della Vita e dell'Ambiente; In *Programma Nazionale Italiano per la Raccolta Dati alieutici 2012*; Università di Cagliari: Cagliari, Italia, 2013. (In Italian)
99. Carbonara, P.; Spedicato, M.T.; Casciaro, L.; Bitetto, I.; Facchini, M.T.; Zupa, W.; Gaudio, P.; Surico, A.; Gallo, P.; Fazi, S.; et al. Campionamento Biologico delle catture. Sezioni C ed E. Rapporto Finale. GSA19 Mar Ionio. In *Programma Nazionale Italiano per la Raccolta Dati alieutici 2012*; COISPA Tecnologia Ricerca: Italia, 2012. (In Italian)
100. De Ranieri, S. Campionamento Biologico delle catture. Sezioni C ed E. Rapporto Finale. In *Programma Nazionale Italiano per la Raccolta Dati alieutici 2011*; Consorzio per il Centro Interuniversitario di Biologia Marina ed Ecologia Applicata "G. Bacci": Italia, 2012. (In Italian)
101. Minervini, R.; Giannotta, M.; Bianchini, M.L. Observations on the fishery of Rajiformes in Central Tyrrhenian Sea. *Oebalia* **1985**, *11*, 583–591.
102. Abella, A.J.; Auteri, R.; Baino, R.; Lazzaretti, A.; Righini, P.; Serena, F.; Silvestri, R.; Volani, A.; Zucchi, A. Reclutamento di forme giovanili nella fascia costiera toscana (Juveniles recruitment along the Tuscan coastal area). *Biol. Mar. Mediterr.* **1997**, *4*, 172–181. (In Italian)
104. Catalano, B.; Dalù, M.; Scacco, U.; Vacchi, M. New biological data on *Raja brachyura* (Chondrichthyes, Rajidae) from around Asinara Island (NW Sardinia, Western Mediterranean). *Ital. J. Zool.* **2007**, *74*, 55–61, doi:10.1080/11250000600831600.
105. Porcu, C.; Bellodi, A.; Cannas, R.; Marongiu, M.F.; Mulas, A.; Follesa, M.C. Life-history traits of a commercial ray, *Raja brachyura* from the central western Mediterranean Sea. *Med. Mar. Sci.* **2014**, *16*, 90–102, doi:10.12681/mms.898.
107. Ramírez-Amaro, S.; Ordines, F.; Terrasa, B.; Esteban, A.; García, C.; Guijarro, B.; Massutí, E. Demersal chondrichthyans in the western Mediterranean: Assemblages and biological parameters of their main species. *Mar. Freshw. Res.* **2016**, *67*, 636, doi:10.1071/mf15093.
108. Barone, M. Life History of *Raja clavata* (Chondrichthyes, Rajiformes). PhD Thesis, Faculty of Mathemathics, Physics and Natural Science of the University of Pisa, Pisa, Italy, 2009.
109. Saglam, H.; Ak, O. Reproductive biology of *Raja clavata* (Elasmobranchii: Rajidae) from Southern Black Sea coast around Turkey. *Helgol. Mar. Res.* **2011**, *66*, 117–126, doi:10.1007/s10152-011-0252-5.
110. Bilgin, S.; Onay, H. Spawning Period and Size at Maturity of the Thornback ray, *Raja clavata* (Linnaeus, 1758), (Elasmobranchii: Rajidae) in the Black Sea. *Acta Aquat. Turc.* **2020**, *16*, 525–534, doi:10.22392/actaquatr.735868.
111. Krstulović Šifner, S.; Vrgoč, N.; Dadić, V.; Isajlović, I.; Peharda, M.; Piccinetti, C. Long-term changes in distribution and demographic composition of thornback ray, *Raja clavata*, in the northern and central Adriatic Sea. *J. Appl. Ichthyol.* **2009**, *25*, 40–46, doi:10.1111/j.1439-0426.2008.01204.x.
112. Carbonara, P.; Bellodi, A.; Palmisano, M.; Mulas, A.; Porcu, C.; Zupa, W.; Donnaloia, M.; Carlucci, R.; Sion, L.; Follesa, M.C. Growth and Age Validation of the Thornback Ray (*Raja clavata* Linnaeus, 1758) in the South Adriatic Sea (Central Mediterranean). *Front. Mar. Sci.* **2020**, *7*, 586094, doi:10.3389/fmars.2020.586094.
113. Ungaro, N. Biological parameters of the brown ray, *Raja miraletus*, in the Southern Adriatic basin. *Cybium* **2004**, *28*, 174–176.
114. Abdel-Aziz, S.H. The use of vertebral rings of the brown ray *Raja miraletus* (Linnaeus, 1758) off Egyptian Mediterranean coast for estimation of age and growth. *Cybium* **1992**, *16*, 121–132.
115. Bellodi, A.; Cau, A.; Marongiu, M.F.; Mulas, A.; Porcu, C.; Vittori, S.; Follesa, M.C. Life history parameters of the small Mediterranean-endemic skate, *Raja polystigma* Regan 1923, from Sardinian seas. In Proceedings of the 5th International Otolith Symposium ICES-CIEM, Mallorca, Spain, 20–24 October 2014; pp. 230–231.

116. Sin, T.M.; Ang, H.P.; Buurman, J.; Lee, A.C.; Leong, Y.L.; Ooi, S.K.; Steinberg, P.; Teo, S.L. Uncommon biological patterns of a little known endemic Mediterranean skate, *Raja polystigma* (Risso, 1810). *Reg. Stud. Mar. Sci.* **2020**, *34*, 101065, doi:10.1016/j.rsma.2020.101065.
117. Yığın, C.Ç.; Işmen, A. Age, growth and reproduction of the rough ray, *Raja radula* (Delaroche, 1809) in Saros Bay (North Aegean Sea). *J. Black Sea/Mediterr. Environ.* **2014**, *20*, 213–227.
118. Tiralongo, F.; Messina, G.; Gatti, R.C.; Tibullo, D.; Lombardo, B.M. Some biological aspects of juveniles of the rough ray, *Raja radula* Delaroche, 1809 in Eastern Sicily (central Mediterranean Sea). *J. Sea Res.* **2018**, *142*, 174–179, doi:10.1016/j.seares.2018.10.001.
120. Yığın, C.Ç.; Işmen, A. Age, growth, reproduction and feed of bottlenosed skate, *Rostroraja alba* (Lacepède, 1803) in Saros Bay, the north Aegean Sea. In Proceedings of the ICES Annual Science Conference, Gdańsk, Poland, 20–24 September 2010; pp. 20–24.
121. Özbek, E.Ö.; Çardak, M.; Kebapçioğlu, T. Spatio-temporal patterns of abundance, biomass and length-weight relationships of *Dasyatis* species (Pisces: Dasyatidae) in the Gulf of Antalya, Turkey (Levantine Sea). *J. Black Sea/Mediterr. Environ.* **2015**, *21*, 169–190.
122. Girgin, H.; Başusta, N. Testing staining techniques to determine age and growth of *Dasyatis pastinaca* (Linnaeus, 1758) captured in Iskenderun Bay, northeastern Mediterranean. *J. Appl. Ichthyol.* **2016**, *32*, 595–601, doi:10.1111/jai.13077.
123. Serena, F. Field identification guide to the sharks and rays of the Mediterranean and Black Sea. In *FAO Species Identification Guide for Fishery Purposes*; FAO: Rome, Italy, 2005; 97p.
124. Yeldan, H.; Avsar, D.; Manaşırlı, M. Age, growth and feeding of the common stingray (*Dasyatis pastinaca*, L., 1758) in the Cilician coastal basin, northeastern Mediterranean Sea. *J. Appl. Ichthyol.* **2009**, *25*, 98–102, doi:10.1111/j.1439-0426.2008.01075.x.
125. Yigin, C.C.; Ismen, A. Age, growth and reproduction of the common stingray, *Dasyatis pastinaca* from the North Aegean Sea. *Mar. Biol. Res.* **2012**, *8*, 644–653, doi:10.1080/17451000.2012.659667.
126. Ismen, A. Age, growth, reproduction and food of common stingray (*Dasyatis pastinaca* L., 1758) in İskenderun Bay, the eastern Mediterranean. *Fish Res.* **2003**, *60*, 169–176, doi:10.1016/s0165-7836(02)00058-9.
127. Saadaoui, A.; Saidi, B.; Enajjar, S.; Bradai, M.N. Reproductive biology of the common stingray *Dasyatis pastinaca* (Linnaeus, 1758) off the Gulf of Gabès (Central Mediterranean Sea). *Cah. Biol. Mar.* **2015**, *56*, 389–396.
128. Başusta, N.; Aslan, E. Age and growth of bull ray *Aetomylaeus bovinus* (Chondrichthyes: Myliobatidae) from the northeastern Mediterranean coast of Turkey. *Cah. Biol. Mar.* **2018**, *59*, 107–114, doi:10.21411/CBM.A.5F77152E.
139. Capapé, C.; Guélorget, O.; Vergne, Y.; Quignard, J.P.; Amor, M.M.B.; Bradaï, M.N. Biological observations on the black torpedo, *Torpedo nobiliana* Bonaparte 1835 (Chondrichthyes: Torpedinidae), from two Mediterranean areas. *Ann. Ser. Hist. Nat.* **2006**, *16*, 19.
140. Barone, M.; De Ranieri, S.; Fabiani, O.; Pirone, A.; Serena, F. Gametogenesis and maturity stages scale of *Raja asterias* Delaroche, 1809 (Chondrichthyes, Raijdae) from the South Ligurian Sea. *Hydrobiologia* **2007**, *580*, 245–254, doi:10.1007/s10750-006-0448-x.
141. Bakiu, R.; Kolitari, J.; Lleshaj, A. Biological Characteristics and Length-Weight Relationships of landed Thornback Ray (*Raja clavata*, Linnaeus 1758) in the Fishing Port of Durres, Albania. *Albanian J. Agric. Sci.* **2021**, *20*, 14–23.
142. Capapé, C.; Quignard, J.P. Contribution ò lo biologie des Rajidae. *Raja polystigma*. *Cah. Biol. Mar.* **1978**, *19*, 233–244.
143. Deval, M.C.; Saygu, İ.; Güven, O.; Özgen, G. Elasmobranch species caught by demersal trawl fisheries in Gulf of Antalya, eastern Mediterranean. In Proceedings of the GFCM First Transversal Expert Meeting on the status of Elasmobranches in the Mediterranean and the Black Sea, Sfax, Tunisia, 20–22 September 2010.
144. Hemida, F.; Seridji, R.; Ennajar, S.; Bradai, M.N.; Collier, E.; Guelorget, O.; Capape, C. New observations on the reproductive biology of the pelagic stingray, *Dasyatis violacea* Bonaparte, 1832 (Chondrichthyes: Dasyatidae) from the Mediterranean Sea. *Acta Adriat.* **2003**, *44*, 193–204.
