# Supplementary material for: Batoid Abundances, Spatial Distribution, and Life History Traits in the Strait of Sicily (Central Mediterranean Sea): Bridging a Knowledge Gap through Three Decades of Survey
Source: Animals (Basel). 2021 Jul 23;11(8):2189. doi: 10.3390/ani11082189 (PMC8388512; doi:10.3390/ani11082189)
Supplement: Supplementary file 1 [file animals-11-02189-s001.zip › Table_S4.pdf]

Class: CHONDRICHTHYES	Scientific name and Authority	Remarks: synonymy and importance to fisheries	MRRL
**Order:** TORPEDINIFORMES			
**Family** TORPEDINIDAE electric rays	*Tetronarce nobiliana* (Bonaparte, 1835)	Electric rays are usually discarded. Rare in the Mediterranean, but widespread distribution. Bycatch in bottom trawl and artisanal demersal fisheries in coastal grounds	LC
	Torpedo marmorata Risso, 1810	Not used for human consumption or other purposes. Taken as bycatch in bottom fisheries but usually discarded or retained for public aquaria. Mediterranean widespread.	LC
	Torpedo sinuspersici Olfers, 1831	Taken as bycatch in demersal trawl and long-line fisheries but usually discarded. Lessepsian species.	NI
	Torpedo torpedo (Linnaeus, 1758)	Taken as bycatch in bottom fisheries but usually discarded.	LC

		Mediterranean widespread.	
**Order:** RHINOPRISTIFORMES			
**Family** RHINOBATIDAE guitarfishes	*Rhinobatos rhinobatos* (Linnaeus, 1758)	*Raja rhinobatos* Linnaeus, 1758. Off the west African coasts, this species is taken as bycatch of international shrimp trawl fleets, bottom trawl cephalopod fisheries, and in artisanal gill net fisheries. In the Mediterranean, it appears more prevalent in the southern and eastern regions.	EN
**Family** GLAUCOSTEGIDAE giant guitarfishes	*Glaucostegus cemiculus* Geoffroy Saint-Hilaire, 1817	*Rhinobatos cemiculus* Geoffroy Saint-Hilaire, 1817. Usually it is a by-catch in bottom trawl fisheries, even if in some areas of the Mediterranean it is caught by gillnet, bottom set, and drifting longlines.	EN
	Glaucostegus halavi (Forsskål, 1775)	*Raja halavi* Forsskål, 1775. A target and utilized bycatch in fisheries. Alien species.	NI

	Pristis pectinata Latham, 1794	*Pristis acutirostris* Duméril, 1865. Artisanal fisheries may take considerable numbers. Rare in the Mediterranean.	CR
**Family** PRISTIDAE sawfish	*Pristis pristis* (Linnaeus, 1758)	*Pristis antiquorum* Latham, 1794. Artisanal fisheries may take considerable numbers as this species is easily taken in shallow waters or often gets entangled in gill nets. Rare in the Mediterranean.	CR
**Order:** RAJIFORMES			
**Family** RAJIDAE rays and skates	*Dipturus batis* (Linnaeus, 1758)	*Raja batis* Linnaeus, 1758. Although never directly targeted, it was taken as bycatch in multispecies bottom trawl fisheries, and it was also taken on long-lines.	CR
	Dipturus nidarosiensis (Storm, 1881)	*Raja nidarosiensis* Storm, 1881. A rare deep water skate taken accidentally in bottom trawl and longline fisheries, occasionally landed.	NI

	Atlantic species.	
Dipturus oxyrinchus (Linnaeus, 1758)	*Raja oxyrinchus* Stehmann, 1970. It was and is a bycatch species in bottom trawl and bottom longline fisheries; often discarded. Widespread in the Mediterranean.	NT
Leucoraja circularis (Couch, 1838)	It has not been subject to target fisheries, but it is taken as a bycatch in bottom trawls and on longlines when usually landed and used for human consumption. Recorded mostly in the western Mediterranean.	CR
Leucoraja fullonica (Linnaeus, 1758)	This skate is mostly taken as bycatch in bottom trawl, gillnet, and longline fisheries targeting other bony fish species. Recorded mostly in the western Mediterranean.	CR
Leucoraja melitensis (Clark, 1926)	*Raja melitensis* Clark, 1926. Although heavily fished, it is also taken in large	CR

	numbers as bycatch in other targeted fisheries. Rare in the Mediterranean, mostly in the west.	
Leucoraja naevus (Muller & Henle, 1841)	*Raja naevus* Stehmann, 1970. Although heavily fished, it is also taken in large numbers as bycatch in other targeted fisheries.	NT
Raja asterias Delaroche, 1809	*Raja punctata* Risso, 1810. It is caught as bycatch by bottom trawl, trammel net, and gill net fisheries especially in the Italian, French, and Spanish seas. Mediterranean Endemic.	NT
Raja brachyura Lafont, 1873	Traditionally landed from multispecies fisheries, although may be targeted in areas of high local abundance, and used for human consumption. Occur in the western Mediterranean.	NT
Raja clavata Linnaeus, 1758	*Raja aspera* Risso, 1810. This species was a target species of	NT

	commercial importance in many demersal fisheries, as well as a regular bycatch. Mediterranean widespread.	
Raja miraletus Linnaeus, 1758	*Raja ocellifera* Regan, 1906. A rather common species, regularly landed along West Africa, and widespread in the Mediterranean.	LC
Raja montagui Fowler, 1910	*Raja punctata* Woodward, 1901. Demersal on soft substrate on shelf from 30 to 150 m depth, rarely as deep as 530 m. Regular bycatch of commercial trawl fisheries, large specimens marketed.	LC
Raja polystigma Regan, 1923	It is part of the bycatch in fisheries using bottom trawl nets, but is also fished with gillnets, longlines, and handlines in artisanal fisheries. Mediterranean endemic.	LC
Raja radula Delaroche, 1809	*Raja atra* Müller &	EN

		Henle, 1841. This skate is commonly taken as bycatch of demersal trawl, gillnet, trammel net, bottom longline, and purse seine fisheries. Mediterranean endemic, more abundant in the western sector.	
	Raja undulata Lacepède, 1802	*Raja fenestra* Rafinesque, 1810. This species is taken as landed bycatch by trawl, trammel net, and other demersal fisheries. Atlantic species.	NT
	Rostroraja alba (Lacépède, 1803)	*Raja alba* Lacepède, 1803b. *R. alba* is captured as bycatch to the multi-species trawl fisheries operating within its range.	EN
**Order:** MYLIOBATIFORMES			
**Family** DASYATIDAE stingray	**Bathytoshia lata* (Garman, 1880)	*Bathytoshia centroura, Dasyatis centroura.* Usually bycatch in trawl, longline, trammel net, and rod and line fisheries,	VU

	but usually discarded.	
**Dasyatis marmorata* (Steindachner, 1892)	Occasionally caught as bycatch of trawl fisheries, but no data are available to determine catch levels or trends.	DD
**Dasyatis pastinaca* (Linnaeus, 1758)	*Raja pastinaca* Linnaeus, 1758. Occasional bycatch in bottom trawl and gillnet fisheries.	VU
**Dasyatis tortonesei* Capapé, 1975	Its taxonomic status is unclear because some authors consider it as synonym of *D. pastinaca*.	NI
**Himantura leoparda* Manjaji-Matsumoto & Last, 2008	*Trygon russellii* Gray, 1834. It is caught by demersal trawl and tangle/gill nets, dropline, and longline fishing and coastal artisanal fisheries (Manjaji and White, 2004). Lessepsian species.	NI
**Himantura uarnak* (Gmelin, 1789)	*Dasybatus uarnak* Gmelin, 1789. It is taken as a utilized bycatch of tangle/gillnet, trawl, and dropline fisheries.	NI

		Lessepsian species.	
	**Pteroplatytrygon violacea* (Bonaparte, 1832)	*Dasyatis violacea* Bonaparte, 1832. It is caught by drift nets and by drifting lines of hooks targeting tuna and swordfish, so it is often discarded at sea because of their low or no commercial value.	LC
	**Taeniura grabata* (Geoffroy Saint-Hilaire, 1817)	*Trygon grabatus* Geoffroy St. Hilaire, 1817. Fishers catch it rarely by trammel nets. Atlantic species.	DD
**Family** GYMNURIDAE butterfly ray	**Gymnura altavela* (Linnaeus, 1758)	*Raja altavela* Linnaeus, 1758. Uncommon in the catch of demersal fisheries trawl and set nets throughout the Mediterranean Sea (mainly found on the southern shores).	CR
**Family** AETOBATIDAE pelagic eagle ray	**Aetomylaeus bovinus* (Geoffroy St. Hilaire, 1817)	*Pteromylaeus bovinus* Geoffroy Saint-Hilaire, 1817. Regularly discarded in mixed species fisheries, locally common.	CR

**Family** MYLIOBATIDAE bull and eagle ray	**Myliobatis aquila* (Linnaeus, 1758)	*Leiobatus aquila* Linnaeus, 1758. Regularly discarded in mixed species fisheries where they are locally common.	VU
**Family** RHINOPTERIDAE cownose ray	*Rhinoptera marginata* (Geoffroy St. Hilaire, 1817)	*Rhinoptera peli* Bleeker, 1863. Regularly discarded in mixed species fisheries, locally common.	DD
**Family** MOBULIDAE devil ray	**Mobula mobular* (Bonnaterre, 1788)	*Manta mobular* Bonnaterre, 1788. More or less regularly an accidental catch in pelagic fisheries with purse seines, trawls, driftnets, and longlines, mostly discarded.	EN

MRRL, Mediterranean Regional Red List; CR, Critically Endangered; EN, Endangered; VU, Vulnerable; NT, Near Threatened; LC, Least Concern; DD, Data Deficient; NI, not included in the MRRL. Asterisk (*) denotes venomous species.
