# Supplementary figures and images for: Batoid Abundances, Spatial Distribution, and Life History Traits in the Strait of Sicily (Central Mediterranean Sea): Bridging a Knowledge Gap through Three Decades of Survey
Source: Animals (Basel). 2021 Jul 23;11(8):2189. doi: 10.3390/ani11082189 (PMC8388512; doi:10.3390/ani11082189)

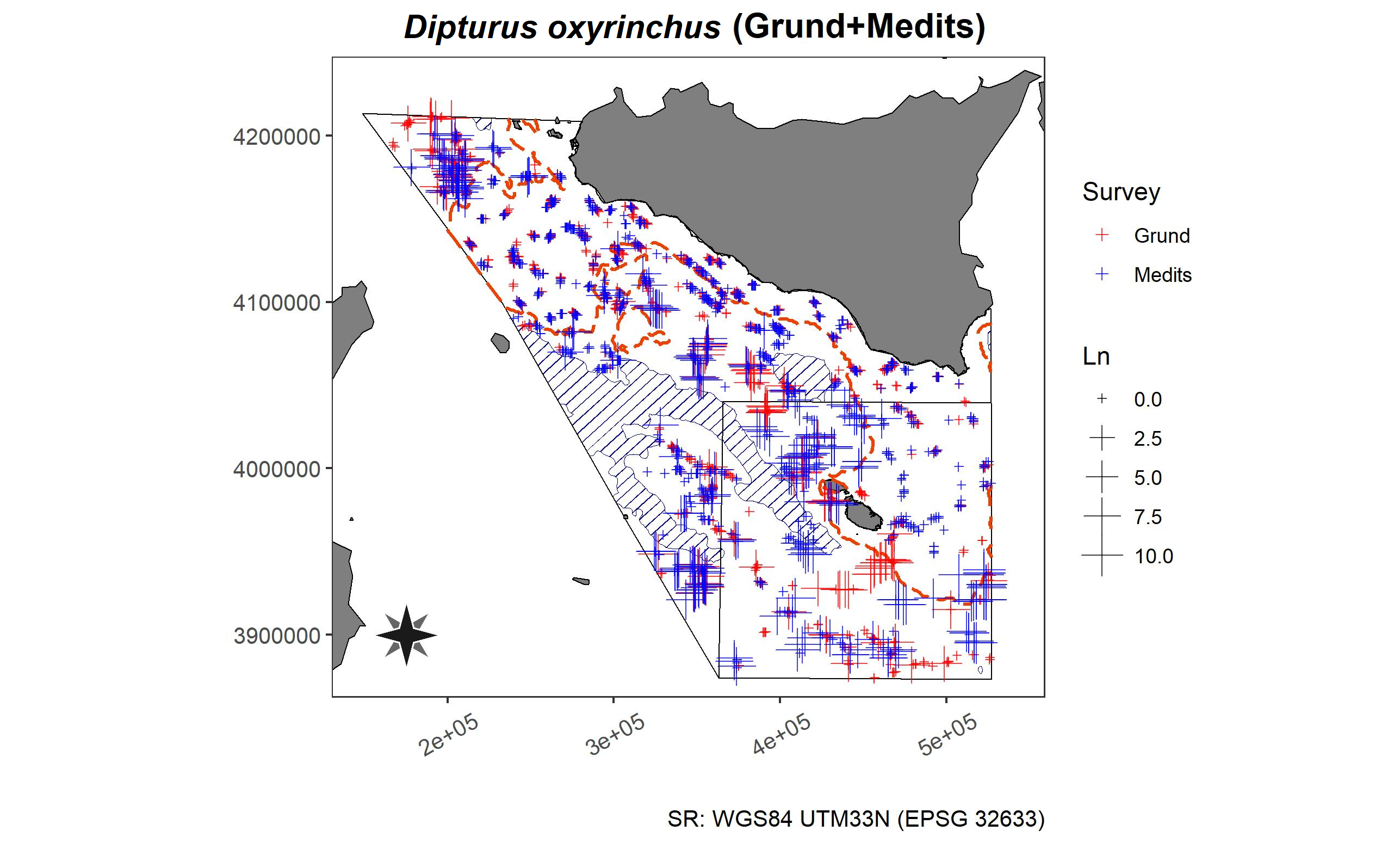

Supplement: Supplementary file 1 [file animals-11-02189-s001.zip › Fig S1.tif]

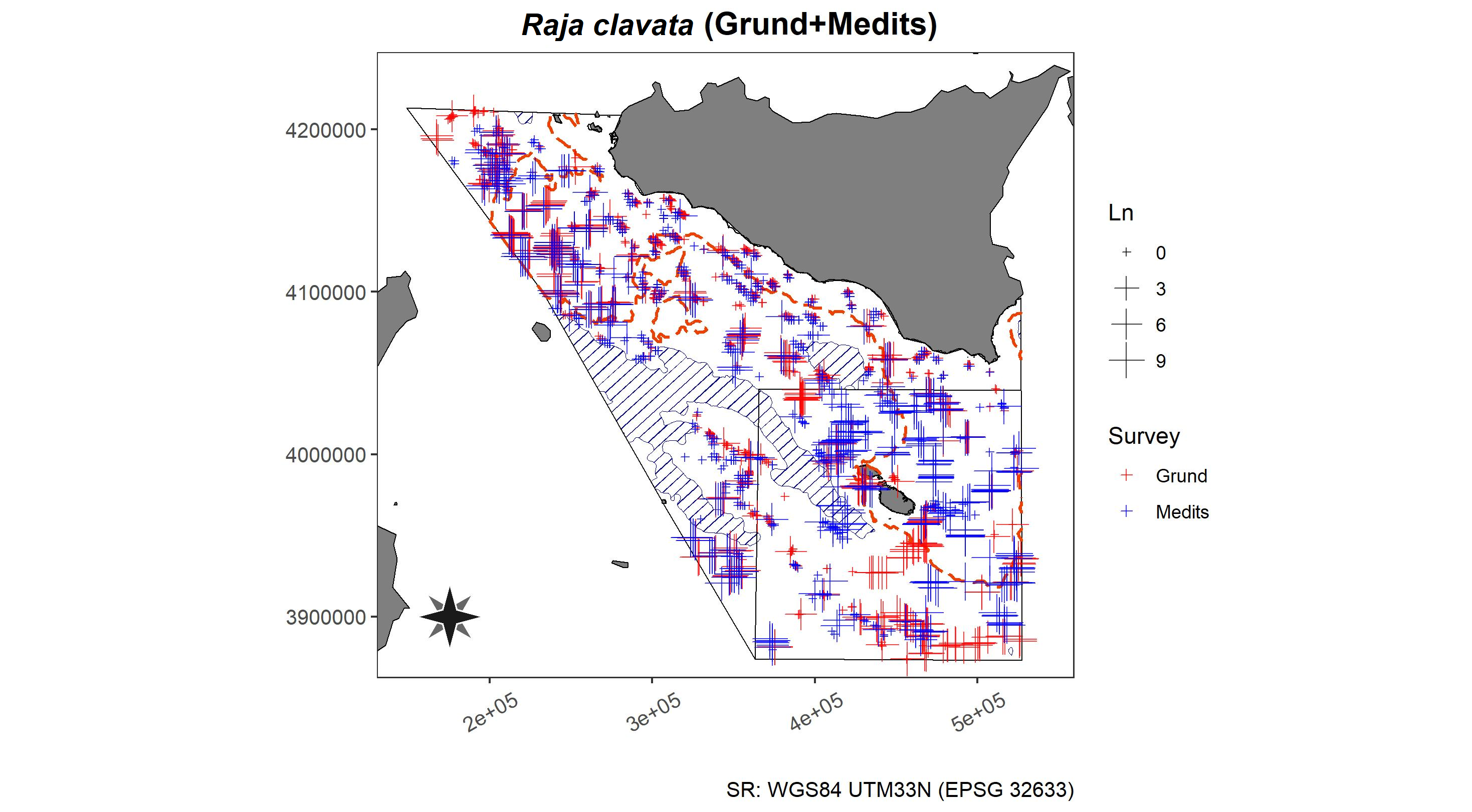

Supplement: Supplementary file 1 [file animals-11-02189-s001.zip › Fig S2.tif]

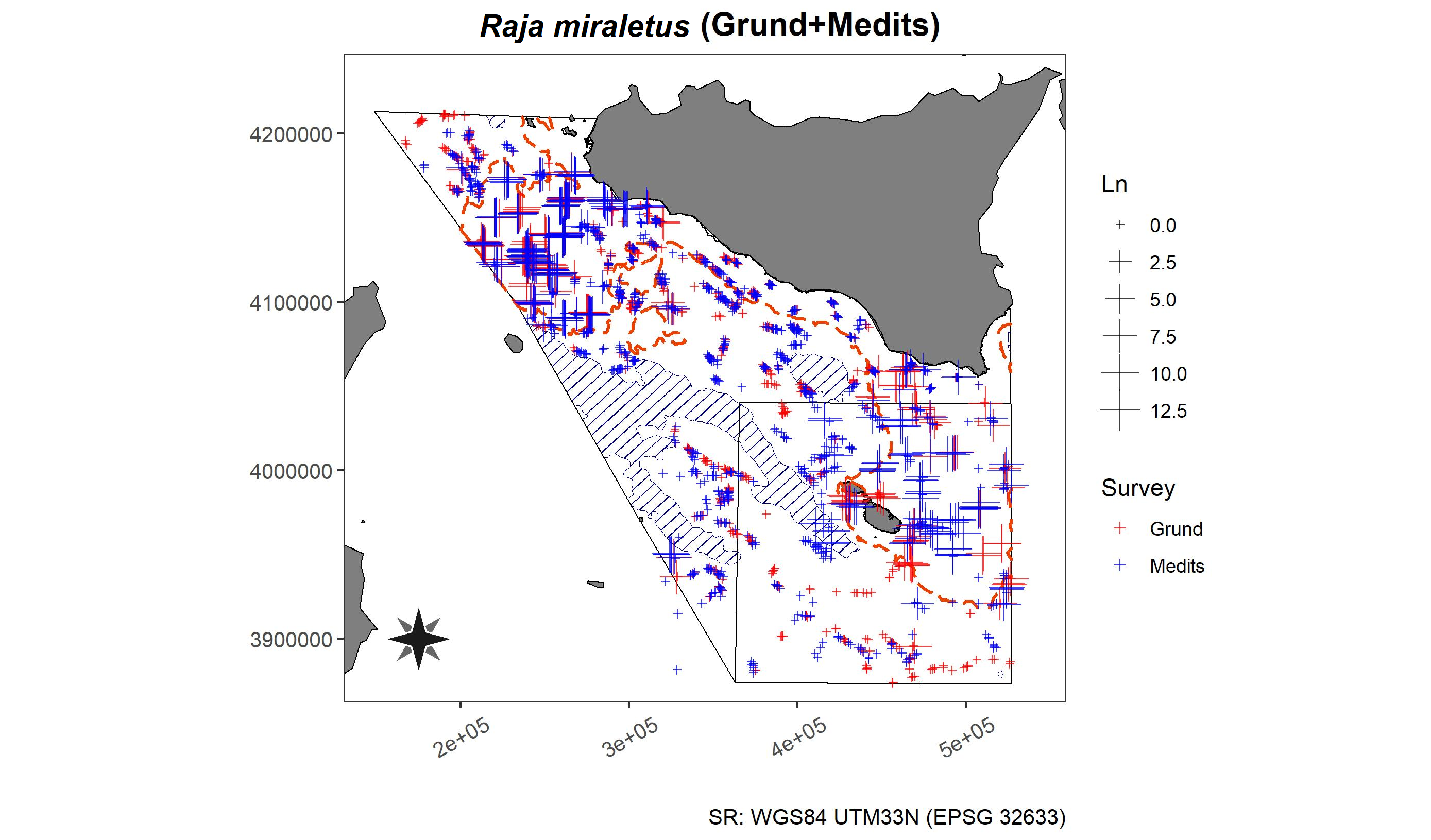

Supplement: Supplementary file 1 [file animals-11-02189-s001.zip › Fig S3.tif]
